# KCC2 downregulation after sciatic nerve injury enhances motor function recovery

**DOI:** 10.1038/s41598-023-34701-y

**Published:** 2023-05-15

**Authors:** Dennis Lawrence Cheung, Takuya Toda, Madoka Narushima, Kei Eto, Chitoshi Takayama, Tatsuko Ooba, Hiroaki Wake, Andrew John Moorhouse, Junichi Nabekura

**Affiliations:** 1 Division of Homeostatic Development, National Institute for Physiological Sciences, Okazaki, Aichi Japan; 2 Department of Physiology, School of Allied Health Sciences, Kitasato University, Sagamihara, Kanagawa Japan; 3 University of the Ryukyus, Nishihara, Okinawa Japan; 4 Division of Multicellular Circuit Dynamics, National Institute for Physiological Sciences, Okazaki, Aichi Japan; 5 Graduate School of Medicine, Nagoya University, Nagoya, Aichi Japan; 6 School of Biomedical Sciences, UNSW Sydney (The University of New South Wales), Sydney, New South Wales Australia; 7 School of Life Science, SOKENDAI (The Graduate University for Advanced Studies), Okazaki, Aichi Japan

**Keywords:** Neuroscience, Physiology

## Abstract

Injury to mature neurons induces downregulated KCC2 expression and activity, resulting in elevated intracellular [Cl^−^] and depolarized GABAergic signaling. This phenotype mirrors immature neurons wherein GABA-evoked depolarizations facilitate neuronal circuit maturation. Thus, injury-induced KCC2 downregulation is broadly speculated to similarly facilitate neuronal circuit repair. We test this hypothesis in spinal cord motoneurons injured by sciatic nerve crush, using transgenic (CaMKII-KCC2) mice wherein conditional CaMKIIα promoter-KCC2 expression coupling selectively prevents injury-induced KCC2 downregulation. We demonstrate, via an accelerating rotarod assay, impaired motor function recovery in CaMKII-KCC2 mice relative to wild-type mice. Across both cohorts, we observe similar motoneuron survival and re-innervation rates, but differing post-injury reorganization patterns of synaptic input to motoneuron somas—for wild-type, both VGLUT1-positive (excitatory) and GAD67-positive (inhibitory) terminal counts decrease; for CaMKII-KCC2, only VGLUT1-positive terminal counts decrease. Finally, we recapitulate the impaired motor function recovery of CaMKII-KCC2 mice in wild-type mice by administering local spinal cord injections of bicuculline (GABA_A_ receptor blockade) or bumetanide (lowers intracellular [Cl^−^] by NKCC1 blockade) during the early post-injury period. Thus, our results provide direct evidence that injury-induced KCC2 downregulation enhances motor function recovery and suggest an underlying mechanism of depolarizing GABAergic signaling driving adaptive reconfiguration of presynaptic GABAergic input.

## Introduction

KCC2 (gene *SLC12A5*) is a neuron-specific isoform of the potassium chloride cotransporter family that acts to maintain low intracellular [Cl^−^]^1,2^. Thus, robust KCC2 activity is critical for safeguarding the efficacy of hyperpolarizing and shunting inhibition mediated by GABAergic (and glycinergic) signaling in neurons, as these depend on the passive inward flux of Cl^−^ through activated ion channels^3^.

In the developing nervous system however, KCC2 expression and activity is low, which results in GABA instead evoking robust membrane depolarization in neurons^4,5^. This is important for various aspects of nervous system development including neuron proliferation, morphological maturation and circuit refinements^6,7^. For example, depolarization of immature neurons by ambient GABA during early development enhances dendritic elongation and branching^8^, interneuron migration in the cerebral cortex^9^ and synaptic efficacy of developing mossy fiber-CA3 connections^10^. Similarly, in the adult brain, depolarization of newly born hippocampal progenitor cells by ambient GABA is critical for their growth and integration into the surrounding neuronal network^11^.

KCC2 activity and expression also becomes downregulated in mature neurons following injury/trauma^12^. This KCC2 downregulation is often transient, generally resolving within weeks^13,14^. In motoneuron axotomy models, loss of KCC2 expression consequent to injury results in elevated resting intracellular [Cl^−^], conversion of GABA-evoked responses to a depolarizing phenotype, and increased frequency of GABA mediated Ca^2+^ transients^13,15^. Notably, the time-course by which KCC2 expression in injured motoneurons gradually recovers towards normal levels parallels that of nerve regeneration^16–18^.

It has frequently been suggested that injury-induced KCC2 downregulation forms part of a general reversion by mature neurons to a quasi-immature phenotype directed towards increasing their capacity to undertake repair of damaged neuronal circuits. This is consistent with both the importance of depolarizing GABA-evoked activity to normal nervous system development, and the close temporal relationship between KCC2 expression and injury resolution in mature neurons. However, the paucity of tools that can selectively manipulate injury-induced KCC2 downregulation has made it difficult to directly test this hypothesis in an in vivo setting—these issues now addressed by this study in two ways.

First, this hypothesis is examined in vivo in spinal cord motoneuron circuits using the sciatic nerve crush (SNC) injury model. SNC immediately impairs motor function by injuring motoneurons at their axons. Importantly, the post-injury motoneuron survival rate is relatively high^19^ and motor function fully recovers after a few weeks^20^. Furthermore, SNC induces a robust decrease in the functionally active pool of plasmalemmal KCC2 in motoneurons^21^. Thus, the SNC injury model serves as a simple in vivo experimental platform for testing whether injury-induced KCC2 downregulation aids or impedes the repair of disrupted spinal cord motoneuron circuits necessary for motor function recovery.

Second, injury-induced KCC2 downregulation is selectively prevented using a transgenic (CaMKII-KCC2) mouse, in which KCC2 expression is additionally coupled to the CaMKIIα promoter^22^. This coupling is facilitated by the tetracycline conditional (tetO-tTA) expression system (Supp. Fig. 1), with the tetracycline transactivator (tTA) gene inserted downstream of the CaMKIIα promoter, and the tetracycline response element (tetO) promoter inserted upstream of the KCC2 gene^23^. In mature motoneurons, CaMKIIα expression is normally very low, but rapidly and strongly increases after axonal injury^24^. Thus, CaMKIIα promoter driven KCC2 expression is minimal in healthy motoneurons, but becomes activated in injured motoneurons and compensates for the downregulation of KCC2 expression that otherwise occurs after SNC.

Overall, this study demonstrates that preventing injury-induced KCC2 downregulation impairs motor function recovery. Furthermore, it associates the impaired recovery with altered GABAergic signaling and reorganization of synaptic inputs to motoneurons. Altogether, this is the most direct evidence to date that injury induced KCC2 downregulation can indeed be causally associated with neuronal circuit adaptations that aid functional recovery after nerve injury.

## Results

### Injury-induced KCC2 downregulation in motoneurons is prevented in CaMKII-KCC2 mice

**Figure 1:**
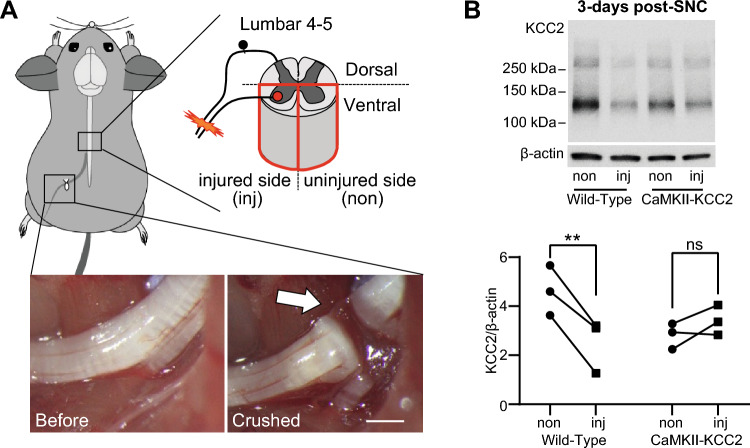
KCC2 downregulation in the spinal cord ventral horn following sciatic nerve crush is prevented in CaMKII-KCC2 mice. (**A**) Schematic and photographs of the sciatic nerve crush (SNC) procedure targeting the left hind-limb in mice. As shown in the spinal cord schematic, SNC only affected motoneurons in the injured-side (left) ventral horn of L4-L5 spinal cord. As shown in the photographs, SNC was performed on the sciatic nerve region proximal to the nerve trifurcation running adjacent to the sciatic notch. The before/after comparison highlights how SNC disconnected the nerve fiber but preserved the epineurium (arrow). Scale bar, 1 mm. (**B**) KCC2 protein expression in L4-L5 ventral horn at 3-days post-SNC for wild-type and CaMKII-KCC2 mice. (**Top**) Representative injured-side (inj) and uninjured-side (non) western blots for KCC2 and β-actin, for both mouse cohorts. The band between the 150 kDa and 100 kDa markers corresponds to the 140 kDa KCC2 monomer. Note that the western blots displayed here have been cropped from the full length western blot in Supp. Fig. 8. (**Bottom**) Each pair of connected data points plots KCC2 protein expression normalized to β-actin for injured-side (inj) and uninjured-side (non) ventral horns from one mouse. Cohorts [wild-type, CaMKII-KCC2], n = [3, 3] mice. *p < 0.05, **p < 0.01; 2-way repeated measures ANOVA, post hoc Bonferroni’s multiple comparisons test.

For all mice, nerve crush (SNC) was performed at the proximal trifurcation of the left sciatic nerve, disconnecting the nerve fiber but leaving the epineurium intact (Fig. 1A). This injured the axons of motoneurons with somas in Rexed lamina IX of the left (injured-side) ventral horn within the L4-L5 spinal cord segment, whilst motoneurons in the right (uninjured-side) ventral horn were unaffected.

In CaMKII-KCC2 mice, withdrawal of doxycycline supplementation enables the CaMKIIα promoter to independently mediate KCC2 expression (Supp. Fig. 1A). For motoneurons, this minimally affects KCC2 expression in healthy cells, but artificially prevents injury-induced downregulation (Supp. Fig. 1B)^24^. Indeed, in western blots of L4-L5 ventral horns, sampled 3-days post-SNC, KCC2 protein expression (normalized to β-actin) was significantly lower in the injured-side (inj) compared to the uninjured-side (non) for wild-type mice, but similar between both sides for CaMKII-KCC2 mice (Fig. 1B). Note that this 3-days post-SNC time-point was selected based on preliminary screening of various post-SNC time-points via RT-qPCR analysis on L4-L5 ventral horns from SNC-injured wild-type mice (Supp. Fig. 2A).

**Figure 2:**
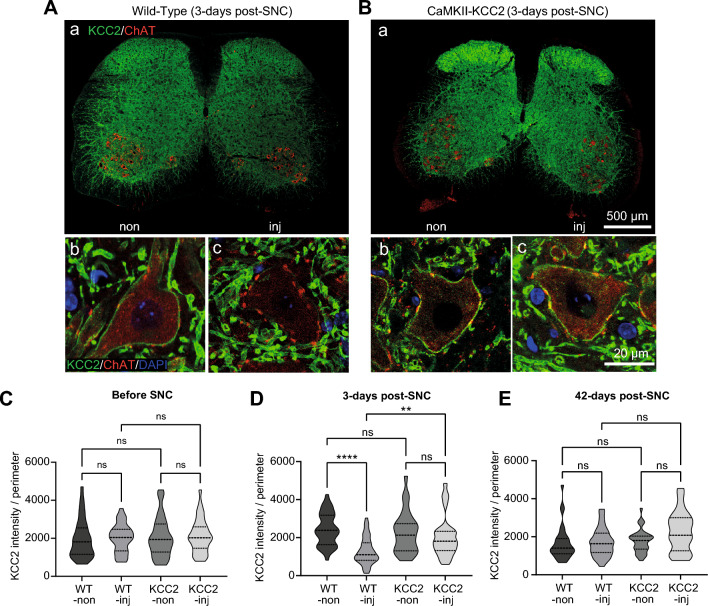
Injury-induced KCC2 downregulation in spinal cord motoneurons is prevented in CaMKII-KCC2 mice. (**A**,**B**) Representative transverse L4-L5 spinal cord sections visualizing KCC2 expression in motoneurons at 3-days post-SNC, from wild-type (**A**: a–c), and CaMKII-KCC2 (**B**: a–c), mice. Immunofluorescence for KCC2 (green), ChAT (red) and DAPI (blue). (**A**: a,**B**: a) Scale bar, 500 μm. At low magnification, red motoneuron somas are clearly localized to Rexed laminae IX in both the injured-side (inj) and uninjured-side (non) ventral horns. (**A**: b,c,**B**: b,c) Scale bar, 20 μm. At high magnification, plasmalemmal KCC2 immunofluorescence in motoneurons is easily visible as the green border along the red soma perimeter. For wild-type mice, plasmalemmal KCC2 immunofluorescence is strong in uninjured-side motoneurons (A: b), but weak in injured-side motoneurons (**A**: c). For CaMKII-KCC2 mice, plasmalemmal KCC2 immunofluorescence is strong in both uninjured-side (**B**: b), and injured-side (**B**: c), motoneurons. (**C**–**E**) Truncated violin plots quantifying plasmalemmal KCC2 immunofluorescence ([plasmalemmal KCC2 intensity]/[soma pixel perimeter]), for individual motoneuron somas from injured-side (inj) and uninjured-side (non) ventral horns (L4-L5, Rexed lamina IX), for wild-type (WT-inj, WT-non), and CaMKII-KCC2 (KCC2-inj, KCC2-non), mice before or at various time-points after SNC. *p < 0.05, **p < 0.01, ***p < 0.001, ****p < 0.0001; Kruskal–Wallis test, post-hoc Dunn’s multiple comparisons test. Cohorts [WT-inj, WT-non, KCC2-inj, KCC2-non]. (**C**) Before SNC, n = [32, 35, 34, 35] cells; 3 wild-type, 3 CaMKII-KCC2 mice. (**D**) 3-days post-SNC, n = [44, 38, 27, 27] cells; 3 wild-type, 2 CaMKII-KCC2 mice. (**E**) 42-days post-SNC, n = [33, 33, 27, 33] cells; 3 wild-type, 3 CaMKII-KCC2 mice.

Only a fraction of cells within the ventral horn are motoneurons, and only KCC2 expressed at the plasma membrane is functionally active. Thus, plasmalemmal KCC2 expression in L4-L5 motoneurons was examined 3-days post-SNC using immunohistochemistry (Fig. 2A,B). For wild-type mice, plasmalemmal KCC2 immunofluorescence was strong in uninjured-side motoneurons (Fig. 2A: b), but weak in injured-side motoneurons (Fig. 2A: c). For CaMKII-KCC2 mice, plasmalemmal KCC2 immunofluorescence was strong in both injured-side and uninjured-side motoneurons (Fig. 2B: b,c).

Subsequently, relative plasmalemmal KCC2 immunofluorescence in L4-L5 motoneurons was quantified ([plasmalemmal KCC2 intensity]/[soma pixel perimeter]) before, 3 and 42-days post-SNC. Individual injured-side (inj) and uninjured-side (non) motoneurons were randomly sampled from wild-type (WT-inj, WT-non) and CaMKII-KCC2 (KCC2-inj, KCC2-non) mice. Before SNC (Fig. 2C) and 42-days post-SNC (Fig. 2E), there were no significant differences between cohorts. In contrast, 3-days post-SNC (Fig. 2D), relative plasmalemmal KCC2 immunofluorescence was significantly lower in WT-inj compared to both WT-non and KCC2-inj, with no significant differences in other pairwise comparisons. Thus injury-induced downregulation of plasmalemmal KCC2 expression occurred in wild-type motoneurons but was prevented in CaMKII-KCC2 motoneurons.

Taken together, these results indicate that SNC induces a transient KCC2 downregulation in wild-type motoneurons which is significant at 3-days post-SNC but resolved by 42-days post-SNC. Importantly, this KCC2 downregulation encompasses a reduction in plasmalemmal KCC2 expression which corresponds to the functionally active KCC2 pool. In contrast, in CaMKII-KCC2 motoneurons, this injury-induced KCC2 downregulation is selectively prevented with no significant distortion of KCC2 expression dynamics in the absence of injury or during the late post-injury period.

### Preventing injury-induced KCC2 downregulation in motoneurons impairs post-injury recovery of motor function

In the SNC injury model, motor function deteriorates immediately post-injury and gradually recovers over the following weeks^25^. Here, post-SNC motor function recovery was tracked using an accelerating rotarod assay. Mice were familiarized with the rotarod task in the week prior to SNC. Motor function was assessed just before (pre), 1, 3, 7, 14, 21 and 42-days post-SNC. The average rotarod speed (rpm, 3 trials) at which mice fell from the rotarod was used as a score of motor function across each assessment day. The relative difference in the pre-SNC and 42-days post-SNC motor scores ([42-days]/[pre] motor score × 100%) was used to gauge the overall extent of motor function recovery.

**Figure 3:**
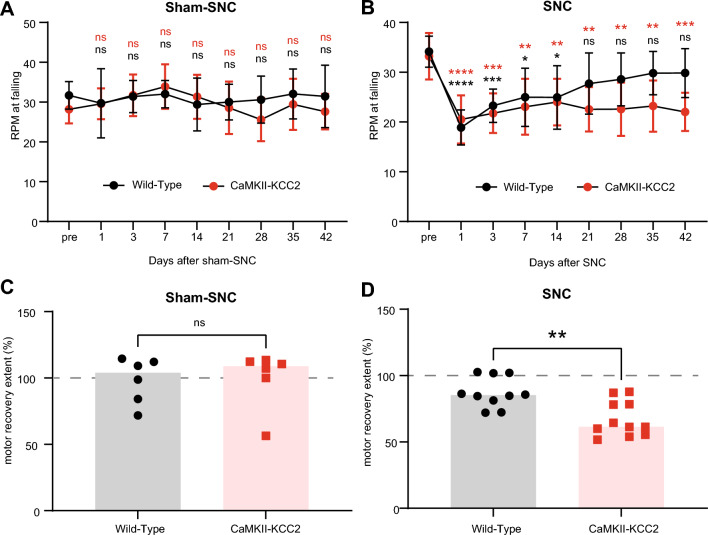
Preventing injury-induced KCC2 downregulation impairs post-injury recovery of motor function. (**A**,**B**) Motor performance scores (rpm at falling), measured before (pre), and at 1, 3, 7, 14, 21, 28, 35 and 42-days after SNC or sham-SNC, for wild-type and CaMKII-KCC2 mice. Cohort means and standard deviations are plotted. *p < 0.05, **p < 0.01, ***p < 0.001, ****p < 0.0001; 2-way repeated measures ANOVA, post hoc Bonferroni multiple comparisons test for pre vs [1, 3, 7, 14, 21, 28, 35, 42]-days post-SNC per each cohort. Cohorts [wild-type, CaMKII-KCC2]. (**A**) Sham-SNC, n = [6, 6] mice. (**B**) SNC, n = [10, 11] mice. (**C**,**D**) Degree of motor function recovery ([day-42/pre] rpm × 100%) after SNC or sham-SNC, for wild-type and CaMKII-KCC2 mice. Each data point represents one mouse. Columns indicate cohort medians. *p < 0.05, **p < 0.01; Mann Whitney test. Cohorts as in (**A**,**B**), respectively. (**C**) Sham-SNC. (**D**) SNC.

To check there was no inherent difference in motor function between wild-type and CaMKII-KCC2 mice, motor function was tracked following a sham-SNC operation (Fig. 3A). Confirming this, motor scores at all time-points remained consistent with pre-SNC levels for both cohorts. Correspondingly, motor recovery extent was essentially complete for both cohorts (Fig. 3C).

Subsequently, to assess the effect of injury-induced KCC2 downregulation on motor function recovery, motor function in wild-type and CaMKII-KCC2 mice was tracked following SNC (Fig. 3B). For both cohorts, motor scores 1-day post-SNC were significantly impaired compared to pre-SNC. For wild-type mice, motor scores reliably recovered to pre-SNC levels from 21-days post-SNC onwards. However, for CaMKII-KCC2 mice, motor scores were still significantly impaired 42-days post-SNC. Consistently, motor recovery extent for CaMKII-KCC2 mice was significantly lower compared to wild-type mice (Fig. 3D). Overall, these results demonstrate that preventing injury-induced KCC2 downregulation in motoneurons impairs motor function recovery.

### Pharmacological blockade of Cl^***−***^ loading in motoneurons post-SNC impairs recovery of motor function

Given KCC2 normally exports Cl^−^, impaired motor function recovery in CaMKII-KCC2 mice could imply that injury-induced KCC2 downregulation benefits injured motoneurons by enabling an elevated intracellular [Cl^−^]. In this case, artificially lowering intracellular [Cl^−^] in SNC-injured motoneurons via a KCC2-independent fashion should similarly impair motor function recovery. To test this hypothesis, NKCC1 activity—the dominant route for Cl^−^ import in most cell types^26^, was blocked in wild-type motoneurons during the early period post-SNC by administering bumetanide, an NKCC1 antagonist. Notably, elevated intracellular [Cl^−^] in axotomized dorsal vagus motoneurons has been correlated with decreased KCC2 expression but unchanged NKCC1 expression that remains functionally active^13^.

**Figure 4:**
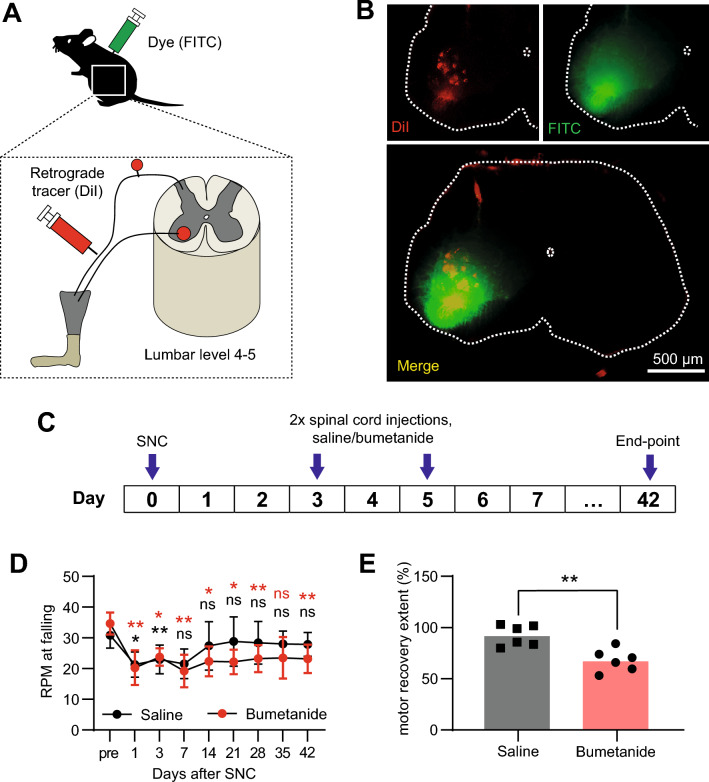
Pharmacological blockade of Cl^−^ loading in motoneurons post-SNC impairs recovery of motor function. (**A**) Schematic summarizing the experimental design used to confirm that injections to the spinal cord at L4-L5 accurately targeted motoneurons whose axons passed through the sciatic nerve. Dye (FITC) was injected into spinal cord at L4-L5 targeting the ventral horn on one side whilst retrograde tracer (DiI) was injected into the ipsilateral sciatic nerve. (**B**) Representative transverse L4-L5 spinal cord section at 7 days after injections into the ventral horn on one side with FITC, and the ipsilateral sciatic nerve with DiI. Overlapping fluorescence of DiI (red) and FITC (green) was localized to the ventral horn ipsilateral to the injected sciatic nerve thus confirming that spinal cord injections could accurately target motoneurons connected to the sciatic nerve. Scale bar, 500 µm. (**C**) Timeline of bumetanide (or saline control) spinal cord injections at 3 and 5-days post-SNC. (**D**) Motor performance scores (rpm at falling), measured before (pre), and at 1, 3, 7, 14, 21, 28, 35 and 42-days after SNC, for wild-type mice administered with saline or bumetanide spinal cord injections at 3 and 5-days post-SNC. Cohort means and standard deviations are plotted. *p < 0.05, **p < 0.01; 2-way repeated measures ANOVA, post hoc Bonferroni multiple comparisons test for pre vs [1, 3, 7, 14, 21, 28, 35, 42]-days post-SNC per each cohort. Cohorts injected with [Saline, Bumetanide], n = [6, 6] mice. (**E**) Degree of motor function recovery ([day-42/pre] rpm × 100%), for wild-type mice administered with saline or bumetanide spinal cord injections at 3 and 5-days post-SNC. Each data point represents one mouse. Columns indicate cohort medians. Cohorts as in (**D**). *p < 0.05, **p < 0.01, Mann Whitney test.

Due to NKCC1’s ubiquitous expression, bumetanide was administered locally via spinal cord injections to avoid potential off-target effects. To confirm this procedure would accurately target SNC-injured motoneurons, FITC dye was injected into the L4-L5 ventral horn on one side whilst DiI dye was injected into the ipsilateral sciatic nerve (Fig. 4A). After 5 days, transverse L4-L5 ventral horn sections were checked for the co-localization of FITC and DiI-positive motoneurons, and restriction of FITC to the injected ventral horn (Fig. 4B).

Subsequently, bumetanide (or saline control) was injected 3 and 5-days post-SNC into the injured-side L4-L5 ventral horn of SNC-injured wild-type mice (Fig. 4C). Using the same rotarod assay, motor function was assessed just before (pre), 1, 3, 7, 14, 21 and 42-days post-SNC (Fig. 4D). For both cohorts, motor scores 1-day post-SNC were again significantly impaired compared to pre-SNC. For saline-treated mice, motor scores reliably recovered to pre-SNC levels from 7-days post-SNC onwards. However, for bumetanide-treated mice, motor scores were still significantly impaired 42-days post-SNC. Consistently, motor recovery extent for bumetanide-treated mice was significantly lower compared to saline-treated mice (Fig. 4E). Overall, these results suggest that reducing Cl^−^ loading in motoneurons during the early post-injury period impairs motor function recovery. Thus, they support the idea that preventing KCC2 downregulation, which subsequently maintains low intracellular [Cl^−^], underpins impaired motor function recovery in CaMKII-KCC2 mice.

### Injury-induced KCC2 downregulation in motoneurons has a limited impact on motoneuron survival

**Figure 5:**
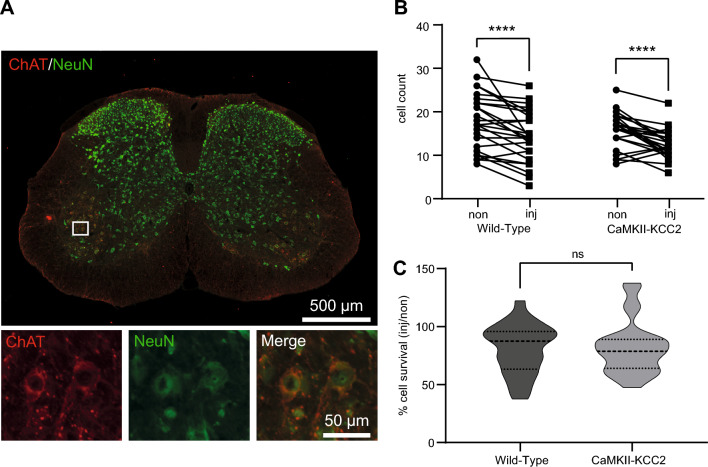
Injury-induced KCC2 downregulation has a limited impact on motoneuron survival. (**A**) Representative transverse L4-L5 spinal cord section showing surviving alpha-motoneurons at 42-days post-SNC. Immunofluorescence for ChAT (red) and NeuN (green). (**Top**) Scale bar, 500 µm. The white box selects an area within Rexed lamina IX in the injured-side ventral horn. (**Bottom**) Scale bar, 50 µm. Magnified view of the selected area above. Alpha-motoneurons co-express ChAT and NeuN. (**B**) Alpha-motoneuron cell counts at 42-days post-SNC in the injured-side (inj) and uninjured-side (non) ventral horn (L4-L5, Rexed lamina IX), for wild-type and CaMKII-KCC2 mice. Each pair of connected data points plots injured-side (inj) and uninjured-side (non) cell counts per one spinal cord section. *p < 0.05, **p < 0.01, ***p < 0.001, ****p < 0.0001; 2-way repeated measures ANOVA, post hoc Bonferroni multiple comparisons test. Cohorts [wild-type, CaMKII-KCC2], n = [25, 26] sections, 3 wild-type, 3 CaMKII-KCC2 mice. (**C**) Truncated violin plots quantifying normalized motoneuron survival ([injured-side/uninjured-side] cell count × 100%) in the injured-side ventral horn (L4-L5, Rexed lamina IX), for wild-type and CaMKII-KCC2 mice at 42-days post-SNC. Cohorts as in (**B**). *p < 0.05; Mann Whitney test.

Given their coupling with the K^+^ (and Na^+^) gradients established by the Na^+^-K^+^-ATPase, Cl^−^ export by KCC2 is highly energetically expensive whilst Cl^−^ import by NKCC1 modestly reduces energy consumption^2^. Thus, impaired motor function recovery in CaMKII-KCC2 mice (and bumetanide-treated wild-type mice) could be due to reduced survival of injured motoneurons, specifically the alpha-motoneuron population innervating muscle fibers. To test this, numbers of L4-L5 alpha-motoneurons (ChAT and NeuN double-positive) in wild-type and CaMKII-KCC2 mice were quantified 42-days post-SNC (Fig. 5A). For both cohorts, alpha-motoneuron counts were modestly but significantly lower in injured-side (inj) compared to uninjured-side (non) (Fig. 5B). Consistently, normalized injured-side alpha-motoneuron survival rate ([injured-side]/[uninjured-side] cell count × 100%) was not significantly different between cohorts (Fig. 5C). Similar patterns were also observed when all motoneuron subtypes were considered (Supp. Fig. 3). Overall, these results suggest that alpha-motoneuron survival rates post-SNC are similar between wild-type and CaMKII-KCC2 mice and thus do not explain impaired motor function recovery in CaMKII-KCC2 mice.

### Injury-induced KCC2 downregulation in motoneurons has a limited impact on the functional re-innervation of downstream muscle targets

**Figure 6:**
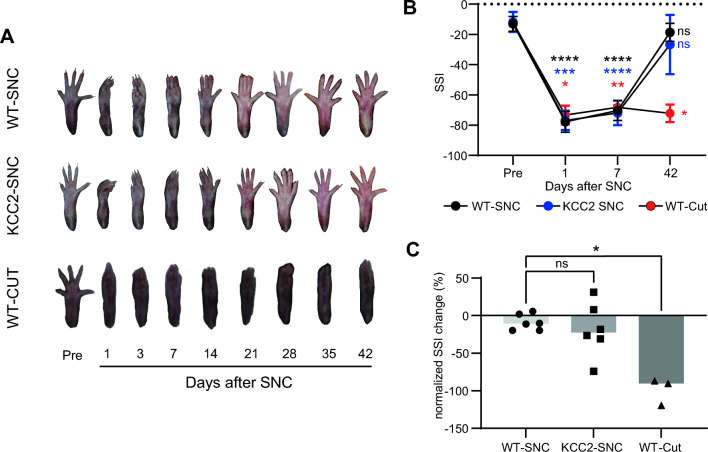
Injury-induced KCC2 downregulation has a limited impact on the functional re-innervation of downstream muscle targets. (**A**) Representative images of the injured-side (left) foot plantar before (pre), and at 1, 3, 7, 14, 21, 28, 35 and 42-days after crush injury (SNC) or complete severing (CUT) of the left sciatic nerve, for wild-type (WT) and CaMKII-KCC2 (KCC2) mice. Measures of toe spread summarized as an SSI score are indicative of sciatic nerve regeneration and downstream muscle re-innervation after injury. (**B**) Periodic SSI scores of the injured-side foot plantar for SNC-injured wild-type (WT-SNC) and CaMKII-KCC2 (KCC2-SNC) mice, or CUT-injured wild-type (WT-CUT) mice. SSI scores were measured before (pre), and at 1, 7 and 42-days after SNC or CUT injury and are plotted as cohort means and standard deviations. Cohorts [WT-SNC, KCC2-SNC, WT-CUT], n = [6, 6, 3] mice. *p < 0.05, **p < 0.01, ***p < 0.001, ****p < 0.0001; 2-way repeated measures ANOVA, post hoc Bonferroni multiple comparisons test for pre vs [1, 7, 42]-days post-SNC per each cohort. (**C**) Normalized SSI change ([(day-42 – pre)/(day-1 – pre)] SSI score x -100%), for SNC-injured wild-type (WT-SNC) and CaMKII-KCC2 (KCC2-SNC) mice and CUT-injured wild-type (WT-CUT) mice. Each data point represents one mouse. Columns indicate cohort medians. Cohorts as in (Fig. 6B). *p < 0.05, Kruskal–Wallis test, post-hoc Dunn’s multiple comparisons test for WT-SNC vs [KCC2-SNC, WT-CUT].

An elevated intracellular [Cl^−^] is associated with accelerated neurite growth in adult sensory neurons after axotomy in vitro^27^. Thus, impaired motor function recovery in CaMKII-KCC2 mice could be due to less efficient axon regeneration and subsequent muscle re-innervation by injured motoneurons. To test this, functional sciatic re-innervation was tracked using sciatic static index (SSI) scores in SNC-injured wild-type (WT-SNC) and CaMKII-KCC2 (KCC2-SNC) mice, and wild-type mice with total sciatic nerve severing and separation to completely block re-innervation (WT-CUT). SSI scores reflected changes in the toe spread of the injured-side hind-paw (Fig. 6A), and have been widely used to quantify the functional recovery of sciatic nerve connections after nerve injury^28,29^. Here, SSI scores were calculated before (pre), 1, 7 and 42-days post-injury (Fig. 6B). For all cohorts, SSI scores 1-day post-injury were significantly deteriorated compared to pre-injury. As expected, SSI scores for WT-CUT mice never recovered, reflecting an absence of any sciatic re-innervation. In contrast, SSI scores for both WT-SNC and KCC2-SNC mice recovered to pre-injury levels by 42-days post-injury. Consistently, normalized SSI recovery ([day-42 – pre]/[day-1 – pre] SSI score × − 100%) for WT-SNC mice was significantly greater than WT-CUT mice but not significantly different to KCC2-SNC mice (Fig. 6C). Overall, these results suggest that post-SNC, functional sciatic re-innervation eventually recovers to a similarly high degree in both wild-type and CaMKII-KCC2 mice and thus does not explain impaired motor function recovery in CaMKII-KCC2 mice.

### Injury-induced KCC2 downregulation in motoneurons can be associated with post-injury reorganization of their synaptic inputs

**Figure 7:**
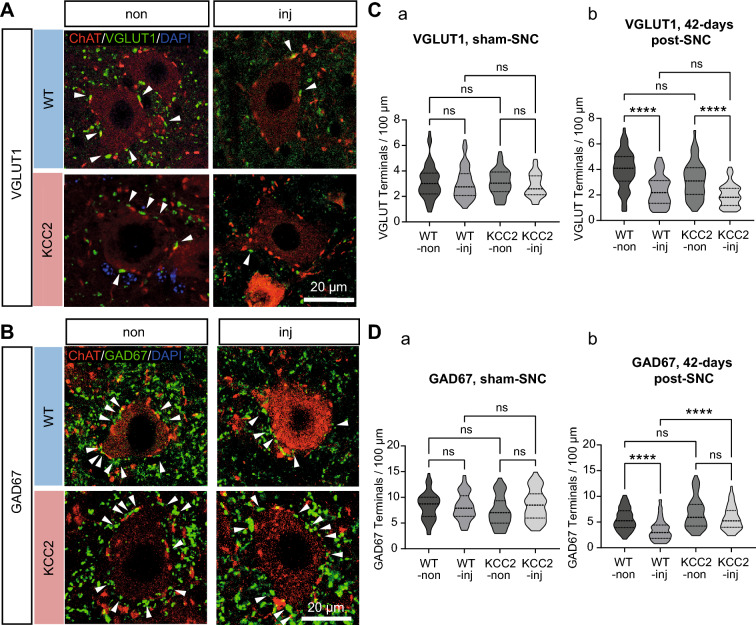
Injury-induced KCC2 downregulation influences post-injury reorganization of motoneuron soma targeting GABAergic (GAD67) terminals but not glutamatergic (VGLUT1) terminals. (**A**,**B**) Representative motoneurons from the injured-side (inj) and uninjured-side (non) ventral horn (L4-L5, Rexed lamina IX), for wild-type (WT) and CaMKII-KCC2 (KCC2) mice at 42-days post-SNC. Immunofluorescence for VGLUT1 or GAD67 (both green, separately), ChAT (red) and DAPI (blue). Scale bar, 20 µm. Terminals synapsing onto the motoneuron soma can be individually resolved as green punctae (arrowheads) along the red soma perimeter. (**A**) VGLUT1 terminals. (**B**) GAD67 terminals. (**C**,**D**) Truncated violin plots quantifying the number of VGLUT1 (C), and GAD67 (D), positive terminals (count per 100 µm), for individual motoneuron somas from the injured-side (inj) and uninjured-side (non) ventral horn (L4-L5, Rexed lamina IX), for wild-type (WT-inj, WT-non), and CaMKII-KCC2 (KCC2-inj, KCC2-non), mice at 42-days after SNC or sham-SNC. *p < 0.05, **p < 0.01, ***p < 0.001, ****p < 0.0001; Kruskal–Wallis test, post-hoc Dunn’s multiple comparisons test. Cohorts [WT-inj, WT-non, KCC2-inj, KCC2-non]. (**C**) VGLUT1 terminal count at: (**C**: a) 42-days after sham-SNC, n = [57, 65, 47, 47] cells, 3 wild-type, 3 CaMKII-KCC2 mice; (**C**: b) 42-days post-SNC, n = [68, 54, 47, 52] cells, 3 wild-type, 3 CaMKII-KCC2 mice. (**D**) GAD67 terminal count at: (**D**: a) 42-days after sham-SNC, n = [46, 56, 50, 43] cells; 3 wild-type, 3 CaMKII-KCC2 mice; (**D**: b) 42-days post-SNC, n = [68, 62, 61, 52] cells, 3 wild-type, 3 CaMKII-KCC2 mice.

Whilst SSI scores reflect resting muscle tone, rotarod performance depends on dynamically synchronizing the activity of all involved muscles. Much of this is coordinated by local spinal cord circuits which undergo significant re-wiring in response to peripheral nerve injury^30^. Thus, impaired motor function recovery in CaMKII-KCC2 mice could be due to maladaptive reorganization of synaptic inputs to motoneurons. To test this, glutamatergic (Fig. 7A) and GABAergic (Fig. 7B) terminals synapsing with L4-L5 motoneuron somas were examined using immunohistochemistry for VGLUT1 and GAD67, respectively. Individual terminals could be resolved as discreet puncta along the motoneuron soma perimeter (Supp. Fig. 4), and were quantified (terminals per 100 µm of soma perimeter). Individual injured-side (inj) and uninjured-side (non) motoneurons were randomly sampled from wild-type (WT-inj, WT-non) and CaMKII-KCC2 (KCC2-inj, KCC2-non) mice.

To check there was no inherent difference in synaptic input organization between wild-type and CaMKII-KCC2 mice, VGLUT1 and GAD67 terminals were quantified 42-days after sham-SNC. Confirming this, there were no significant differences between cohorts for VGLUT1 (Fig. 7C: a) and GAD67 (Fig. 7D: a).

Subsequently, VGLUT1 and GAD67 terminals were quantified 42-days post-SNC. For VGLUT1, terminal counts were significantly lower in WT-inj compared to WT-non, and KCC2-inj compared to KCC2-non, with no significant differences in other pairwise comparisons (Fig. 7C: b). Thus, for both wild-type and CaMKII-KCC2 mice, injured-side motoneurons had significantly fewer VGLUT1 terminals compared to uninjured-side motoneurons. For GAD67, terminal counts were significantly lower in WT-inj compared to both WT-non and KCC2-inj, with no significant differences in other pairwise comparisons (Fig. 7D: b). Thus, for wild-type mice, injured-side motoneurons had significantly fewer GAD67 terminals compared to uninjured-side motoneurons. Whereas for CaMKII-KCC2 mice, injured-side and uninjured-side motoneurons retained similar numbers of GAD67 terminals. Taken together, these results suggest that impaired motor function recovery in CaMKII-KCC2 mice is underpinned, at least in part, by excessive preservation of GABAergic synaptic input to motoneurons.

### Injury-induced KCC2 downregulation promotes motor function recovery through its effect on GABAergic signaling

**Figure 8:**
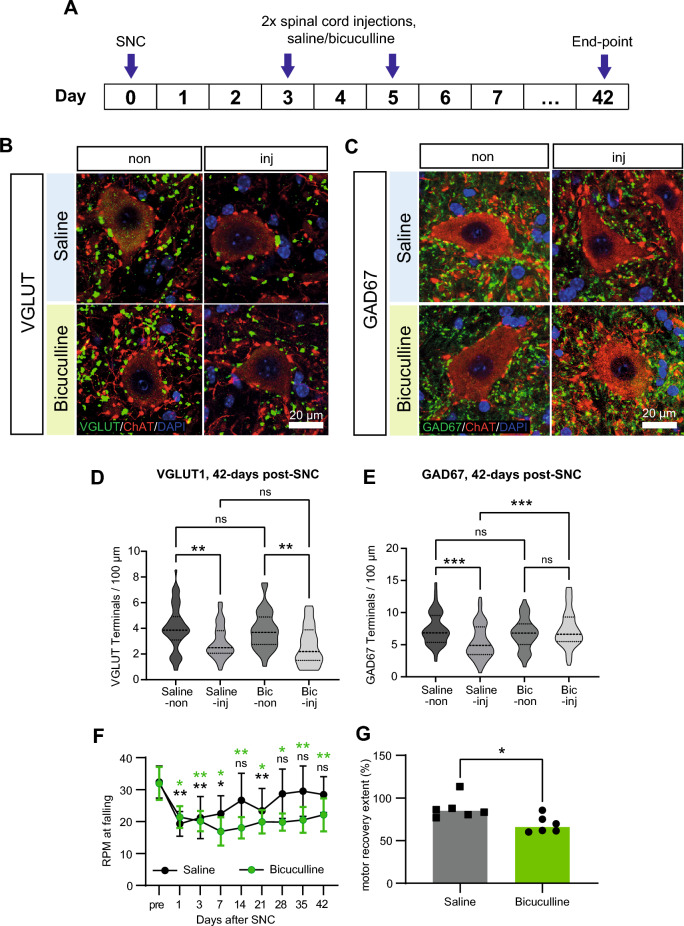
Injury-induced KCC2 downregulation promotes motor function recovery through its effect on GABAergic signaling. (**A**) Timeline of bicuculline (or saline control) spinal cord injections at 3 and 5-days post-SNC. (**B**,**C**) Representative motoneurons from the injured-side (inj) and uninjured-side (non) ventral horn (at L4-L5, Rexed lamina IX), for saline and bicuculline treated wild-type mice at 42-days post-SNC. Drugs administered via spinal cord injection at 3 and 5-days post-SNC. Immunofluorescence for VGLUT1 or GAD67 (both green, separately), ChAT (red) and DAPI (blue). Scale bar, 20 µm. (**B**) VGLUT1 terminals. (**C**) GAD67 terminals. (**D**,**E**) Truncated violin plots quantifying the number of VGLUT1 (**E**), and GAD67 (**F**), positive terminals (count per 100 µm), for individual motoneuron somas from the injured-side (inj) and uninjured-side (non) ventral horn (at L4-L5, Rexed lamina IX), for saline (Saline-inj, Saline-non), and bicuculline (Bic-inj, Bic-non), treated wild-type mice at 42-days post-SNC. Drugs administered via spinal cord injection at 3 and 5-days post-SNC. *p < 0.05, **p < 0.01, ***p < 0.001, ***p < 0.0001; Kruskal–Wallis test, post-hoc Dunn’s multiple comparisons test. Cohorts [Saline-inj, Saline-non, Bic-inj, Bic-non]. (**D**) VGLUT1 terminal count, n = [46, 51, 35, 33] cells, 3 saline, 3 bicuculline mice. (**E**) GAD67 terminal count, n = [62, 61, 53, 46], 3 saline, 3 bicuculline mice. (**F**) Motor performance scores (rpm at falling), measured before (pre), and at 1, 3, 7, 14, 21, 28, 35 and 42-days after SNC, for wild-type mice administered with saline or bicuculline spinal cord injections at 3 and 5-days post-SNC. Cohort means and standard deviations are plotted. *p < 0.05, **p < 0.01; 2-way repeated measures ANOVA, post hoc Bonferroni multiple comparisons test for pre vs [1, 3, 7, 14, 21, 28, 35, 42]-days post-SNC per each cohort. Cohorts injected with [Saline, bicuculline], n = [6, 6] mice. (**G**) Degree of motor function recovery ([day-42/pre] rpm × 100%), for wild-type mice administered with saline or bicuculline spinal cord injections at 3 and 5-days post-SNC. Each data point represents one mouse. Horizontal bars indicate cohort medians. Cohorts as in (**F**). *p < 0.05, **p < 0.01, Mann Whitney test.

The above experiments associate impaired motor function recovery with preventing injury-induced KCC2 downregulation in motoneurons (Figs. 1, 2, 3), reducing intracellular Cl^−^ loading during the early post-injury period (Fig. 4), and the long-term inappropriate preservation of GABAergic synaptic input to motoneurons (Fig. 7). Thus, a key question is whether GABAergic synapse pruning is induced by intracellular Cl^−^ loading itself or via its effect on GABAergic signaling. To address this, GABAergic signaling was blocked in wild-type motoneurons during the early post-injury period following SNC by locally administering bicuculline, a GABA_A_ receptor antagonist. Thus, bicuculline (or saline control) was injected 3 and 5-days post-SNC into the injured-side L4-L5 ventral horn of SNC-injured wild-type mice (Fig. 8A).

Subsequently, glutamatergic (Fig. 8B) and GABAergic (Fig. 8C) terminals synapsing with L4-L5 motoneuron somas were quantified 42-days post-SNC using immunohistochemistry for VGLUT1 and GAD67, respectively. Individual injured-side (inj) and uninjured-side (non) motoneurons were randomly sampled from saline-treated (Saline-inj, Saline-non) and bicuculline-treated (Bic-inj, Bic-non) mice. For VGLUT1, terminal counts were significantly lower in Saline-inj compared to Saline-non, and Bic-inj compared to Bic-non, with no significant differences in other pairwise comparisons (Fig. 8D). For GAD67, terminal counts were significantly lower in Saline-inj compared to both Saline-non and Bic-inj, with no significant differences in other pairwise comparisons (Fig. 8E). Thus, the post-SNC reorganization of VGLUT1 and GAD67 terminals in CaMKII-KCC2 mice was replicated in bicuculline-treated wild-type mice.

Separately, motor function was assessed just before (pre), 1, 3, 7, 14, 21 and 42-days post-SNC using the same accelerating rotarod assay (Fig. 8F). For saline-treated and bicuculline-treated mice, motor scores 1-day post-SNC were again significantly impaired compared to pre-SNC. For saline-treated mice, motor scores reliably recovered to pre-SNC levels from 28-days post-SNC onwards. However, for bicuculline-treated mice, motor scores were still significantly impaired 42-days post-SNC. Consistently, motor recovery extent for bicuculline-treated mice was significantly lower compared to saline-treated mice (Fig. 8G). Thus, impaired motor function recovery in CaMKII-KCC2 mice was replicated in bicuculline-treated wild-type mice.

Overall, these results demonstrate that blocking GABAergic signaling in SNC-injured wild-type mice replicates the phenotype of SNC-injured CaMKII-KCC2 mice. This suggests that injury-induced KCC2 downregulation in motoneurons acts through depolarized GABAergic signaling to remove excessive GABAergic synaptic input during the early post-injury period and thus facilitate motor function recovery.

## Discussion

### Injury-induced KCC2 downregulation promotes motor function recovery

Neuronal circuit repair by mature neurons is thought to rely on their reversion to a quasi-immature phenotype. Presumably, this confers an enhanced capacity for neuroplasticity similar to the developing nervous system where there is extensive formation and pruning of synaptic connections. Depolarizing GABAergic signaling underpinned by low KCC2 expression are notable features of this developmental phenotype, and closely linked with various aspects of neuronal circuit formation and maturation. Thus, injury-induced KCC2 downregulation and the resulting depolarizing GABAergic signaling are often hypothesized to promote neuronal circuit repair, though this idea has not been directly tested until now.

This study confirms this hypothesis for the sciatic nerve crush (SNC) injury model. Injury-induced KCC2 downregulation promotes motor function recovery (Figs. 1, 2, 3) by facilitating adaptive reorganization of disrupted neuronal circuits (Fig. 7). Importantly, CaMKII-KCC2 mice exhibit long-term impairment in their post-injury motor function which confirms that injury-induced KCC2 downregulation is not merely auxiliary to the recovery process but is in fact a key driver. More broadly, the pharmacological experiments with bumetanide (Fig. 4) and bicuculline (Fig. 8) suggest that it is the high intracellular [Cl^−^] that results from KCC2 downregulation and subsequent depolarizing GABAergic signaling that is mechanistically important.

Crucially however, these conclusions only extend to KCC2 downregulation during the early post-injury period. In the SNC model used here, KCC2 expression in injured wild-type motoneurons is significantly reduced at 3-days post-SNC, but is normal at earlier and later time-points (Fig. 2; Supp. Fig. 2). Correspondingly, in injured CaMKII-KCC2 motoneurons, reduced KCC2 expression at this early post-injury time-point is selectively prevented but there is no KCC2 overexpression either prior injury or during the late post-injury period. (Figs. 1B, 2; Supp. Figs. 1, 2). Similarly, bumetanide (Fig. 4) and bicuculline (Fig. 8) would only have significant efficacy close to their administration time at 3 and 5-days post-SNC. Indeed, significant KCC2 downregulation (and depolarizing GABAergic signaling) either occurring acutely (whilst the insult is ongoing) or chronically (late post-injury period) appears to exacerbate the pathological process in other injury models—see below.

Whilst SNC would induce some acute KCC2 downregulation, its impact is not examined in this study. Acute KCC2 downregulation is underpinned by altered phosphorylation patterns, oligomerization states and membrane internalization dynamics^31^, which are not detected by RT-qPCR (Supp. Fig. 2) and would proceed similarly in injured wild-type and CaMKII-KCC2 motoneurons. Pertinently, SNC is a comparatively mild injury, associated with a relatively high motoneuron survival rate^19^, and is not inherently self-escalating, i.e. it is not accompanied by a vicious circle of hyperactive neuronal firing. Thus, acutely compromised GABAergic inhibition is unlikely to be a major contributor to SNC injury severity. This contrasts with more severe injury models such as in vitro neuronal excitotoxicity. Here, blocking acute depolarizing GABAergic signaling reduces injury-induced damage^32^, whilst acute KCC2 expression knockdown further decreases cell viability^33^. Similarly, diazepam potentiation of GABAergic signaling more reliably suppresses protracted seizure activity in vivo when combined with prophylactic KCC2 overexpression^22^.

In addition, SNC does not cause long-term KCC2 downregulation (Fig. 2; Supp. Fig. 2). Whilst the underlying mechanism is unclear, the eventual return to normal KCC2 expression in motoneurons post-injury appears related to axon regeneration^16,17^, and successful re-innervation of downstream muscle targets^18^. In contrast, locomotor spasticity caused by direct spinal cord injury (SCI-spasticity) is associated with chronic KCC2 downregulation^34^, and responds positively to drugs which increase KCC2 expression^35^. At the same time, owing to the more extensive and complex injury, neuronal circuits remain permanently damaged in SCI-spasticity unlike in SNC. Indeed, silencing of surviving neuronal circuits appears to be a key pathophysiological aspect for SCI-spasticity as chronic KCC2 downregulation also occurs in inhibitory neurons and treatment of this alone promotes functional recovery^36^.

### Targeted prevention of injury-induced KCC2 downregulation in motoneurons using transgenic CaMKII-KCC2 mice

A key innovation of this study is the selective prevention of injury-induced KCC2 downregulation in vivo, specifically in spinal cord motoneurons from CaMKII-KCC2 mice. Through coupling via the tetracycline conditional (teto-tTA) expression system (Supp. Fig. 1A), the CaMKIIα promoter provides an additional independent means for driving KCC2 expression, which can be reversibly disabled by doxycycline (tetracycline analogue) supplementation. Thus, by supplementing CaMKII-KCC2 mice with doxycycline from birth (in utero) until 2 to 3-weeks before starting experiments, perturbation of KCC2 expression during development and its associated impacts on nervous system maturation are avoided^23,26^.

For CaMKII-KCC2 spinal cord motoneurons specifically, doxycycline removal alone does not induce KCC2 overexpression (Supp. Fig. 1B). This is because in mature motoneurons, basal transcription of CaMKIIα is very low and only increases after injury—specifically a twofold increase in CaMKIIα mRNA expression when measured 4-days after SNC^24^. Thus, CaMKIIα promoter driven KCC2 expression should be minimal in healthy motoneurons, but active in SNC-injured motoneurons. In this way, SNC injury elegantly triggers both downregulation of KCC2’s natural expression systems and compensatory activation of CaMKIIα promoter driven KCC2 expression.

This assumption that off-doxycycline CaMKII-KCC2 mice have normal KCC2 expression in motoneurons at time-points outside of the early post-injury period is supported by the KCC2 immunohistochemistry experiments (Fig. 2). Whilst subtler basal KCC2 overexpression might be missed, its inconsequence is indicated by the similar rotarod performance (Fig. 3A,C), VGLUT1 terminal (Fig. 7C: a), and GAD67 terminal (Fig. 7D: a) phenotypes of wild-type and CaMKII-KCC2 mice following sham-SNC. Similarly, the influence of subtle KCC2 overexpression during the late post-injury period on the post-SNC phenotype of CaMKII-KCC2 mice is likely minimal for several reasons. First, bumetanide (Fig. 4) and bicuculline (Fig. 8) administration limited to 3 and 5-days post-SNC replicates the post-SNC phenotype of CaMKII-KCC2 mice (Figs. 3B,D, 7C: b,D: b). Second, motor function recovery in CaMKII-KCC2 mice is rescued by delaying doxycycline removal to 14-days post-SNC, i.e. CaMKIIα promoter driven KCC2 expression is only activated during the late post-injury period (Supp. Fig. 6). Finally, when VGLUT1 and GAD67 terminals are examined 7-days post-SNC, their reorganization patterns already greatly resemble those observed 42-days post-SNC (Supp. Fig. 7).

An important caveat of this study is that whilst KCC2 manipulations in motoneurons are restricted to preventing injury-induced KCC2 downregulation, KCC2 overexpression would have occurred upon doxycycline removal in neurons which normally express CaMKIIα. These include excitatory cortical and hippocampal neurons^23^; and potentially some sensory neurons with inputs to the spinal cord, although in this case CaMKIIα is mainly expressed in nociceptive C-fibers that do not directly project to motoneurons^37,38^. Whilst this issue could be circumvented by combining tetO-tTA coupling with motoneuron-specific promoters such as Hb9, Isl1 and ChAT^39^, these lack the injury-correlated time-course of CaMKIIα expression, necessitating careful optimization in timing the removal and re-introduction of doxycycline supplementation.

Notably, Nakamura et al.^40^ previously report that the CaMKII-KCC2 mice used here, show enhanced motor learning as assessed by a rotarod assay. The apparent contradiction with this study however, can be explained by important differences in experimental design. In Nakamura et al.^40^, motor performance is assessed over a successive 5-day period, 6 trials per day, in mice that are initially naïve to the rotarod task. Whereas here, motor performance is assessed intermittently over a 42-day period, 3 trials per day, in (expert) mice that have already been familiarized with the rotarod task during the week before SNC. Thus, the assay design in Nakamura et al.^40^ is tailored to foster and capture early motor learning changes whereas the assay design used here examines established baseline motor performance. This is supported by the sham-SNC rotarod experiments, as motor scores for both wild-type and CaMKII-KCC2 mice are similar and do not significantly change over time (Fig. 3A,C). Furthermore, the bumetanide (Fig. 4) and bicuculline (Fig. 8) experiments affirm the overwhelming importance of spinal cord processes on motor function recovery post-SNC. Nevertheless, it would be interesting to see replication of these data using localized gene transfection of the CaMKII-KCC2 coupled expression system to the spinal cord, although optimizing such approaches for reliable expression whilst minimizing inflammation is technically challenging for motoneurons.

### Possible mechanisms linking KCC2 downregulation in motoneurons to enhanced motor function recovery

Synaptic stripping is a well-characterized sequela to sciatic nerve injury describing a significant generalized loss of presynaptic terminal inputs to motoneuron somas^41,42^. In this study, the differing synaptic stripping patterns at 42-days post-SNC of wild-type vs CaMKII-KCC2 (and bicuculline-treated) mice neatly correlate with their divergent motor recovery outcomes (Figs. 3, 7). For wild type mice, both VGLUT1 and GAD67 injured-side terminal counts are significantly reduced compared to uninjured-side terminal counts. For CaMKII-KCC2 (and bicuculline-treated) mice, this trend is repeated for VGLUT1 terminal counts, whereas GAD67 injured-side and uninjured-side terminal counts are not significantly different.

In the spinal cord ventral horn, VGLUT1 predominantly labels excitatory glutamatergic terminals from muscle afferents^43,44^, whilst GAD67 labels inhibitory GABAergic terminals from local interneurons that are likely activated by both sensory and descending inputs^45^. Motoneurons also receive inhibitory input from glycinergic terminals but GlyT2 immunohistochemistry suggests these do not undergo significant reorganization following SNC (Supp. Fig. 5). For injured-side VGLUT1 terminals at 42-days post-SNC, the scale of loss in wild-type, CaMKII-KCC2 (and bicuculline-treated) mice, is comparable with the ~ 30% loss rate previously reported^46^. Functionally, this implies a substantial reduction in excitatory input to motoneurons given that a ~ 50% loss of VGLUT1 terminals corresponds to the innervation rate of homonymous motoneurons by a single Ia afferent dropping from 90% normally, to < 20%^43,44,47^. Separately, for injured-side GAD67 terminals at 42-days post-SNC, their loss in wild-type mice vs their preservation in CaMKII-KCC2 (and bicuculline-treated) mice implies differing resulting dynamics for GABAergic inhibition. One possibility is that in CaMKII-KCC2 (and bicuculline-treated) mice, the excitatory-inhibitory balance of synaptic drive onto motoneurons becomes overly biased towards inhibition thus resulting in suboptimal motoneuron-muscle coordination and impaired motor function (Supp. Fig. 9).

How injury-induced KCC2 downregulation contributes to synaptic stripping can be linked to its facilitating of a depolarizing shift in GABA-evoked responses. In cultured immature hippocampal neurons, depolarizing GABAergic signaling is associated with long-term depression of both GABAergic^48^, and glutamatergic synapses^49^, whilst precocious KCC2 overexpression facilitates a relative increase in GABAergic but not glutamatergic synapses^50^. Notably, CaMKII-KCC2 mice still show significant VGLUT1 synaptic stripping at 42-days post-SNC (Fig. 7A), which would appear to partially contradict this idea. However, terminal counts at earlier time-points indicate delayed VGLUT1 synaptic stripping in CaMKII-KCC2 mice (Supp. Fig. 7), which is presumably rectified by later acting KCC2-independent VGLUT1 synaptic stripping mechanisms such as microglia-mediated removal of inactive quiescent VGLUT1 terminals^51,52^. Indeed, there is growing appreciation that synaptic stripping is underpinned by multiple, mechanistically distinct processes that are active at different time periods post-injury^42^.

Whilst a limitation of this study is that the degree of depolarization in GABA-evoked responses was not directly measured, this can be inferred approximately from the extent of KCC2 downregulation. In this study, SNC induced a ~ 40% reduction in plasmalemmal KCC2 expression as measured by immunohistochemistry (Fig. 2). This has previously been directly correlated with a ~ 50% reduction in oligomeric plasmalemmal KCC2 expression (the functionally active KCC2 pool), as measured by surface biotinylated western blot^34^. Based on modelling, just a 10–20% reduction in plasmalemmal KCC2 expression in motoneurons reduces the inhibitory efficacy of soma-located GABAergic input by ~ 40%, and converts distally-located GABAergic input from being inhibitory, to facilitating excitatory activity^34^.

All of this would result in an increased frequency of Ca^2+^ transients in motoneurons, underpinned by enhanced NMDA receptor activation due to greater permissiveness, or even directly triggered by GABA itself which is observed in motoneurons following axotomy^13,15^. Regarding GAD67 terminals, this could trigger dispersion and loss of GABA_A_ receptor clusters, similar to the calcineurin-dependent mechanism characterized in cultured hippocampal neurons^53^. Consistently, local blockade of depolarized GABAergic signaling during the early post-injury period facilitates long-term preservation of GAD67 terminals at injured-side motoneurons (Fig. 8). Regarding VGLUT1 terminals, predicted effects are more speculative, though it is likely that the dynamics of GABA-related Ca^2+^ transients will differ to those evoked by glutamate. Thus, an important consideration is that whether increases in intracellular [Ca^2+^] cause strengthening or weakening of glutamatergic synapses depends on their amplitude and duration^54^.

Separately, it should be noted that independent to its Cl^−^ export function, KCC2 also regulates the expression dynamics of other plasmalemmal receptors through direct interactions with the actin cytoskeleton and associated scaffolding proteins. Indeed, loss of plasmalemmal KCC2 destabilizes AMPA receptor clusters through interactions with the actin cytoskeleton^55^, whilst knockdown of the GABA_A_ receptor scaffolding protein gephyrin also reduces plasmalemmal KCC2 expression^56^. For VGLUT1 synaptic stripping, blockade of KCC2-independent synaptic stripping processes (discussed above) is required before the contribution of such mechanisms can be properly assessed. For GAD67 synaptic stripping, the contribution of such mechanisms is likely minor as the similar phenotypes in CaMKII-KCC2, bumetanide-treated and bicuculline-treated mice implicate altered intracellular [Cl^−^], leading to depolarizing GABAergic signaling, as the primary driver.

## Conclusion

Using a transgenic mouse, we leveraged the injury-dependent dynamics of CaMKIIα expression in motoneurons to selectively prevent injury-induced KCC2 downregulation following SNC in vivo. Thus, we directly demonstrate that injury-induced KCC2 downregulation during the early post-injury period truly benefits the functional restoration of spinal cord motoneuron circuits as reflected by improved motor function recovery. At a mechanistic level, we associate this with the removal of excessive GABAergic synaptic input and suggest that this is facilitated by a temporary depolarizing shift in GABAergic signaling induced by transient KCC2 downregulation.

## Materials and methods

### Animals

All procedures were performed at the National Institute for Physiological Sciences which is a constituent institute of the National Institutes for Natural Sciences. All procedures were approved by the Animal Care and Use Committee of the National Institutes of Natural Sciences. All procedures were performed in accordance with all relevant guidelines and regulations as well as ARRIVE guidelines.

The CaMKII-KCC2 transgenic mouse line was established by crossing CaMKII-tTA mice with tetO-KCC2 mice. The CaMKII-tTA mice were a kind gift from Dr. Kenji Tanaka. In these mice, the tetracycline trans-activator (tTA) gene is knocked-in downstream of the CaMKIIα promoter^57^. The tetO-KCC2 mice were generated as described in Goulton et al.^23^. In these mice, the tetracycline response element (tetO) promoter is knocked-in upstream of the KCC2 gene.

The newly generated CaMKII-KCC2 mice were backcrossed with C57BL/6J mice for 10 generations to establish the CaMKII-KCC2 mouse colony used in this study. To prevent excessive colony inbreeding, C57BL/6J mice were periodically introduced into the colony mating scheme. In experiments with a CaMKII-KCC2 cohort, the control wild-type cohort was derived from transgene double-negative (tTA^−^/tetO^−^) littermates. In experiments without a CaMKII-KCC2 cohort, all mice were derived from a pure-bred C57BL/6J mouse colony.

Male mice were used for all experiments and were 8 to 9-weeks old at the time of SNC surgery. After weaning and sex separation, mice were housed in groups of 4–6 animals per cage under a 12-h light–dark cycle with ad libitum access to food and water. In experiments without a CaMKII-KCC2 mouse cohort, mice were raised on standard rodent chow (Oriental Yeast Co). In experiments with a CaMKII-KCC2 cohort, both CaMKII-KCC2 mice and wild-type mice (transgene double-negative littermates) were raised on 100 mg/kg doxycycline supplemented rodent chow (Oriental Yeast Co) until 2 to 3 weeks before surgery, when they were then switched onto standard rodent chow (Supp. Fig. 1B). The only exception to this schedule was in the experiment examining the time-critical nature of injury-induced KCC2 downregulation where the on-doxycycline to off-doxycycline switch was delayed to 14-days after SNC surgery (Supp. Fig. 6A).

### Sciatic nerve crush (SNC)

Mice were 8 to 9-weeks old and weighed 25–30 g at the time of surgery. Ketamine (70 mg/kg) and xylazine (10.5 mg/kg) were used for anaesthesia and administered as a cocktail via intraperitoneal injection. All surgical procedures targeted the left sciatic nerve, specifically the region proximal to the nerve trifurcation which ran adjacent to the sciatic notch. For sciatic nerve crush (SNC), the target region was crushed twice with forceps for 20 s^20^. Importantly, this procedure disconnected the nerve fiber but left the epineurium intact. For sham-SNC, the target region was exposed without crushing. For total severing of the sciatic nerve, the target region was cut with surgical scissors and the nerve stumps separated by 1 mm to prevent axon re-connection.

### Accelerating rotarod assay

Motor function in mice was assessed using an accelerating rotarod assay. Mice were required to balance on a rotating rod (rotarod) by walking/running in sync with the rotarod’s rotational speed, which was gradually increased. The rotarod’s speed (rpm) at which mice fell from the rotarod reflected their level of motor function and was thus recorded as their motor score. All mice were trained daily on the accelerating rotarod assay throughout the week preceding the SNC operation.

On motor function assessment days, the day’s motor score was calculated from the average of three rotarod assay trials, each trial separated by an interval of at least 5 min. For each assay trial, the rotarod (LEB200, Panlab, diameter, 30 mm) was accelerated from 4 to 40 rpm, in increments of 1 rpm over 5 min, with each successive speed maintained for approximately 8 s. The assay trial was stopped after 5 min and a per trial motor score of 40 rpm was assigned to any mice which had not yet fallen off the rotarod.

### Real time RT-qPCR

Mice were deeply anaesthetized with ketamine/xylazine and perfused with 10 mM Ca(II)-EDTA in PBS. Lumbar spinal cords (L4-L5) were harvested and injured-side and uninjured-side ventral horns dissected out. Tissue was homogenized in QIAzol Lysis Reagent (79306, QIAGEN), centrifuged with chloroform, and the supernatant collected. Total RNA was isolated using a RNeasy Plus Universal Mini Kit (73404, QIAGEN) and reverse-transcribed using a Transcriptor First Strand cDNA Synthesis Kit (04379012001, Roche). Real time qPCR was performed using a LightCycler 96 system (Roche) with custom primer sets and SYBR Green Master Mix (FastStart Essential DNA Green Master kit, 06402712001, Roche). mRNA expression levels were calculated using the comparative CT method with GAPDH as the housekeeper gene.

Custom primers used are summarized in Table 1.

**Table 1:** Primers used for RT-qPCR.

Gene	Primer	Sequence
*GAPDH*	Forward	AATGCATCCTGCACCACCAAC
	Reverse	TGGATGCAGGGATGATGTTCTG
*KCC2*	Forward	GGCATTCTTCCAGGCAGTAG
	Reverse	CCCTAATTGGTGTCGATGCT

### Western blot

Mice were deeply anaesthetized with ketamine/xylazine and decapitated. Lumbar spinal cords (L4-L5) were harvested and injured-side and uninjured-side ventral horns dissected out. Tissue was homogenized in ice cold buffer [50 mM Tris–HCl (pH 7.5), 150 mM NaCl, 5 mM EDTA, 1% Triton, protease inhibitor (11697498001, Roche)], centrifuged (12,000×*g*, 10 min, 4 °C), and the supernatant collected. Total protein concentration was determined using a Pierce BCA Protein Assay Kit (23225, Thermo Fisher). Proteins were electrophoretically separated in SDS–polyacrylamide gel (Mini-PROTEAN TGXTM 7.5%, 456–1024, Bio-Rad) and transferred to polyvinylidene fluoride membranes. Membranes were successively incubated in 5% BSA in 0.1 M phosphate buffer (1 h, room temperature), primary antibody (overnight, 4 °C), and horseradish peroxidase conjugated secondary antibody (1 h, room temperature). Protein bands were visualized via chemical luminescence using Luminata TM Forte Western HRP Substrate (Merck Millipore), photographed using a CCD camera, and their optical densities quantified using ImageJ (version 1.47v, National Institute of Health, Bethesda, MD, USA) software. Note that whole KCC2 expression was quantified (glycosylated monomer ~ 140 kDa, SDS resistant non-dissociated dimer ~ 300 kDa)^58^.

Antibody characteristics and dilutions used are summarized in Tables 2, 3.

**Table 2:** Primary antibodies for western blot.

Immunogen		Host and type	Dilution	Source
*Β-actin*	Modified peptide corresponding to the mouse β-actin N-terminus	Mouse monoclonal	1 : 1000	030M4788, Sigma-Aldrich
*KCC2*	N-terminal His-tag fusion protein corresponding to residues 932–1043 of rat KCC2	Rabbit polyclonal	1: 1000	07-432, Chemicon

**Table 3:** Secondary antibodies for western blot.

Target and host	Conjugant	Dilution	Source
Anti-mouse, from goat	Horseradish peroxidase	1 : 1000	HAF007, R&D systems
Anti-rabbit, from goat	Horseradish peroxidase	1 : 5000	HAF008, R&D systems

### Immunohistochemistry staining

Mice were deeply anaesthetized with ketamine/xylazine and perfused with 4% paraformaldehyde (PFA) in 0.1 M phosphate buffer (PB). Lumbar spinal cords (L4-L5) were harvested, post-fixed in 4% PFA (overnight, 4 °C), and stored in 30% sucrose for cryoprotection (> 3 days, 4 °C). Transverse sections (20 μm thick) were prepared using a frozen stage sliding microtome (SM2010R, Leica), collected by free floating in 0.1 M PB, and plated onto gelatin coated glass slides.

For ChAT, NeuN, VGLUT1, GAD67 and VP16 immunohistochemistry, the plated sections were blocked with 5% BSA/0.3% Triton in phosphate buffered saline (PBS) (1 h, room temperature), and incubated with primary antibodies (overnight, 4 °C/room temperature for ChAT). For KCC2 immunohistochemistry, the plated sections were dried, then successively incubated in methanol (2 × 15 min), HCl (10 min, 37 °C), pepsin (S3002, Dako; 2 min, 37 °C), and primary antibody (overnight, room temperature)^59^.

The next day, the plated sections were washed with PBS, incubated with Alexa Fluor conjugated secondary antibody (2 h, room temperature), and coverslipped with fluorescence protecting mounting medium (Vector Laboratories).

Antibody characteristics and dilutions used are summarized in Tables 4–5.

**Table 4:** Primary antibodies for immunohistochemistry.

Immunogen		Host and type	Dilution	Source
*ChAT*	Purified protein from human placenta	Goat polyclonal	1:250	AB144P, Chemicon
*GAD67*	Purified protein from mouse, amino acids 87–106	Mouse monoclonal	1:500	G5419, SIGMA
*KCC2*	Synthetic peptide, N-terminal of mouse KCC2, amino acids 1022–1042	Rabbit polyclonal	1:1000	Original antibody^59^
*NeuN*	Purified protein from mouse brain cell nuclei brain	Mouse monoclonal	1:500	MAB377, Chemicon
*VGLUT1*	Purified recombinant protein, rat VGLUT1, amino acids 456–560	Guinea pig polyclonal	1:500	135–304, Synaptic Systems

**Table 5:** Secondary antibodies for immunohistochemistry.

Target and host	Conjugant	Dilution	Source
Anti-goat, from donkey,	Alexa Fluor 594	1:500	A-11058, ThermoFisher
Anti-guinea pig, from goat	Alexa Fluor 488	1:500	A-11073, ThermoFisher
Anti-mouse, from goat	Alexa Fluor 488	1:500	A-11001, ThermoFisher
Anti-rabbit, from donkey	Alexa Fluor 488	1:500	A-21206, ThermoFisher

### Histological quantification

Transverse spinal cord sections (20 µm thick) were imaged using a confocal laser scanning microscope (A1, Nikon) with single photon excitation wavelengths of 488 and 594 nm, and a 60 × objective lens (PlanApo-VC 60xA WI, NA = 1.20). Images were collected as 3D stacks spanning 20 µm in the Z-axis with successive optical sections separated by 0.4 μm.

Histological quantification was performed on randomly selected ChAT-positive neurons in ventral horn Rexed laminae IX of the L4-L5 spinal cord segment as these correspond to motoneurons which innervate the distal lower limb muscles via the sciatic nerve^43^. ImageJ (Version 1.47v, National Institute of Health, Bethesda, MD, USA) was used for all quantification procedures. Information concerning the genotype and injured-side vs uninjured-side identity of selected neurons was concealed before histological quantification to facilitate blinded and unbiased analysis.

Double counting of motoneurons was prevented as follows. To avoid double counting in the XY-plane, the borders of adjacent image stacks were checked to confirm there was no overlap. To avoid double counting in the Z-axis, the total number of motoneuron nuclei (ChAT immunofluorescence channel) in each image stack was counted by scrolling through the entire series of optical sections. The image stack was then cropped (XY-plane) and split (Z-axis) into sub-stacks which each contained only one motoneuron. To avoid double counting due to a motoneuron being split between two separate histological sections, only sub-stacks which captured the appearance and disappearance of the motoneuron nucleus (ChAT immunofluorescence channel) within its series of optical sections were used for analysis.

For each motoneuron (image sub-stack), image analysis used the 3–4 successive optical sections which captured the motoneuron nucleus. An ROI demarcating the motoneuron soma border was initially defined using the ChAT immunofluorescence of the centre-most optical section. This was then visually checked against the ChAT immunofluorescence of the other adjacent optical sections. In all instances, this check indicated that no modifications to the ROI were required. Essentially, there was no significant change in the shape/size of the motoneuron soma across the narrow Z-distance as defined by the stack of 3–4 optical sections. Subsequently, the 3–4 optical sections were combined into a single image using the maximum intensity projection function in ImageJ. The resulting image was used for all image analysis procedures.

Plasmalemmal KCC2 expression in motoneurons was quantified as follows. The soma perimeter as demarcated by ChAT immunofluorescence was traced (described above) thus defining two categories of KCC2-positive pixels. KCC2-positive pixels inside the ROI were considered as cytosolic KCC2 whilst KCC2-positive pixels along the ROI were considered as (enriched for) plasmalemmal KCC2. Background subtraction was performed by calculating the average brightness (0–255 grayscale) of KCC2-positive pixels within the ROI and subtracting this value across the entire image (KCC2 immunofluorescence channel). This essentially zeroed-out all cytosolic KCC2 signal whereas strong plasmalemmal KCC2 signal was still visible. Thus, the average cytosolic KCC2 brightness was used as the threshold for selecting KCC2-positive pixels along the ROI, i.e. plasmalemmal KCC2. The grayscale values of these KCC2-positive pixels along the ROI were then summed to give a single value that represented the total plasmalemmal KCC2 signal intensity of the analyzed motoneuron. To account for variations in motoneuron size, the plasmalemmal KCC2 signal intensity value was divided by the motoneuron pixel perimeter length.

VGLUT1-positive and GAD67-positive terminals synapsing onto motoneuron somas could be resolved as distinct punctae along the soma perimeter and were quantified as follows. The soma perimeter as demarcated by ChAT immunofluorescence was traced (described above) to generate a line plot profile of VGLUT1 or GAD67 immunofluorescence intensity (Supp. Fig. 4). From this fluorescence intensity plot, peaks larger than 2 × the baseline and wider than 2 µm were counted as individual VGLUT1/GAD67-positive terminals. To account for variations in motoneuron size, the VGLUT1/GAD67-positive terminal counts were divided by the motoneuron soma perimeter length and expressed as terminal density per 100 μm of soma perimeter.

GlyT2-positive terminals synapsing onto motoneuron somas were unable to be resolved as distinct punctae and were instead quantified as follows. Background GlyT2 immunofluorescence was eliminated by a threshold automatically calculated from the entire field of view. A soma perimeter ROI was defined by tracing around the motoneuron soma as demarcated by ChAT immunofluorescence (described above) and the number of GlyT2-positive pixels along the ROI was counted. To account for variations in motoneuron size, the GlyT2-positive pixel count was divided by the total soma perimeter ROI pixel area and expressed as a percentage.

### Sciatic static index (SSI)

Post-injury sciatic nerve regeneration and its re-innervation of downstream muscle targets was assessed using the sciatic static index (SSI), calculated from plantar measurements of both the injured-side and uninjured-side hind-paws. Mice were placed in a transparent corridor and allowed to freely walk about. Once they had settled to a standing and stationary posture, their hind-paws were photographed (Canon iVIS HF R800 camera) through the transparent floor. From these photographs, the paw length (PL) and the distance between the first and fifth toe (TS) were measured, and their values entered into the SSI formula described in Baptista et al.^28^:

$$\begin{aligned} SSI{\text{ }} = & \left( {\left[ {{\text{injured}} - {\text{side TS}}} \right]{\mkern 1mu} - {\mkern 1mu} \left[ {{\text{uninjured}} - {\text{side TS}}} \right]} \right){\mkern 1mu} \\ & \times 101.3/\left[ {{\text{uninjured}} - {\text{side TS}}} \right] - \left( {\left[ {{\text{injured}} - {\text{side PL}}} \right] - \left[ {{\text{uninjured}} - {\text{side PL}}} \right]} \right){\mkern 1mu} \\ & \times 54.03/\left[ {{\text{uninjured}} - {\text{side PL}}} \right] - 9.5. \\ \end{aligned}$$

### Local injection of drugs into the spinal cord

Mice were anesthetized with isoflurane (4% induction, 1–2% maintenance) and kept on a warming pad during surgery. The spinal cord was exposed by blunt dissection. Muscle tendons connecting the spinal column were cut to isolate the spinous and superior articular processes of the Th13 and L1 vertebral bodies which cover the L4-L6 spinal cord segment^60^. Muscle tendons at all other levels of the spinal column were left intact. The spinal column was then secured in a custom made surgical frame and a small laminectomy was performed on the Th13 and L1 vertebral bodies to partially expose the left side of the L4-L6 spinal cord segment (ipsilateral to the injured sciatic nerve). A glass pipette (20 μm tip diameter) was slowly inserted 1.0–1.2 mm below the exposed spinal cord dura surface to target the left ventral horn and 500 nl of saline or drugs (20 μM bicuculline or 50 μM bumetanide, Sigma Aldrich) were pressure injected (7–10 psi, IM 300 Microinjector, Narishige). The pipette was kept in place for approximately 10 min after completing the injection and then removed. Subsequently, the paraspinous muscles were sutured over the laminectomized spinal column and the skin incision closed with surgical staples.

To confirm that this surgical procedure accurately targeted motoneurons whose axons passed through the sciatic nerve, FITC dye (Sigma Aldrich) mixed with saline was injected into the ventral horn on one side (as described above) whilst DiI dye (Sigma Aldrich) mixed with ethanol was injected into the ipsilateral sciatic nerve. Transverse spinal cord sections were prepared 1 week later to check for dye co-localization and restriction to the targeted area. This was performed as a separate experiment.

### Statistical analysis

All statistical tests were performed using GraphPad Prism 9. In all cases, hypothesis testing was two-tailed with the alpha significance level set to 0.05. For some datasets, the only available statistical test was parametric, specifically the 2-way repeated measures ANOVA. In these cases, datasets were confirmed to be normally distributed by Q-Q plots of the ANOVA residuals. For all other datasets, non-parametric statistical tests were used.

The statistical tests used and sample sizes are described in the figure legends. The exact calculated p-values for each figure in the main text are summarized in the Supplementary Data.

## Supplementary Information

Supplementary Figures.

## Data Availability

All data associated with this study is available upon reasonable request. This should be made to the corresponding author through the provided email address.
