# Supplementary material for: KCC2 downregulation after sciatic nerve injury enhances motor function recovery
Source: Sci Rep. 2023 May 15;13:7871. doi: 10.1038/s41598-023-34701-y (PMC10185507; doi:10.1038/s41598-023-34701-y)
Supplement: Supplementary file 1 — Supplementary Figures. [file 41598_2023_34701_MOESM1_ESM.pdf]

KCC2 downregulation after sciatic nerve injury enhances motor function recovery

Dennis Lawrence Cheung[1], Takuya Toda[1], Madoka Narushima[1], Kei Eto[1], Chitoshi Takayama[2], Tatsuko Ooba[1], Hiroaki Wake[1], Andrew John Moorhouse[3], Junichi Nabekura[1]*

1. The National Institute for Physiological Sciences, Japan; 2. University of the Ryukyus, Japan; 3. UNSW Sydney, Australia

Corresponding author: Junichi Nabekura, nabekura@nips.ac.jp

Supplementary Data: Summary of Contents

Page	Item
2	Statistics Summary: Figure 1
3	Statistics Summary: Figure 2
4	Statistics Summary: Figure 3
5	Statistics Summary: Figure 4
6	Statistics Summary: Figure 5
7	Statistics Summary: Figure 6
8	Statistics Summary: Figure 7
9	Statistics Summary: Figure 8
10	Supplementary Figure 1
11	Supplementary Figure 2
12	Supplementary Figure 3
13	Supplementary Figure 4
14	Supplementary Figure 5
15	Supplementary Figure 6
17	Supplementary Figure 7
19	Supplementary Figure 8
21	Supplementary Figure 9

Statistics Summary: Figure 1.

**1B**			
2-way repeated measures ANOVA	**F (DFn, DFd)**	**p-value**	**significance**
**genotype x inj/non**	F (1, 4) = 32.03	0.0048	**
**genotype**	F (1, 4) = 0.5070	0.5158	n/a
**inj/non**	F (1, 4) = 10.12	0.0335	n/a
**subject**	F (4, 4) = 7.438	0.0388	*
Post hoc Bonferroni multiple comparisons test		**adjusted p-value**	**significance**
**non vs inj**	**Wild-Type**	0.0067	**
	**CaMKII-KCC2**	0.3089	ns
Sample size	**n, L4-L5 ventral horn samples**		**n, mice**
**Wild-Type (inj)**	3		3
**Wild-Type (non)**	3		
**CaMKII-KCC2 (inj)**	3		3
**CaMKII-KCC2 (non)**	3		

Statistics Summary: Figure 2.

	2C		2D		2E	
Kruskal-Wallis test	**p-value**	**significance**	**p-value**	**significance**	**p-value**	**significance**
	0.7721	ns	<0.0001	****	0.0374	*
Post hoc Dunn's multiple comparisons test	**adjusted p-value**	**significance**	**adjusted p-value**	**significance**	**adjusted p-value**	**significance**
**WT-non vs WT-inj**	>0.9999	ns	<0.0001	****	>0.9999	ns
**KCC2-non vs KCC2-inj**	>0.9999	ns	>0.9999	ns	0.5733	ns
**WT-non vs KCC2-non**	>0.9999	ns	>0.9999	ns	0.6720	ns
**WT-inj vs KCC2-inj**	>0.9999	ns	0.0086	**	0.1143	ns
Sample size	**n, cells**	**n, mice**	**n, cells**	**n, mice**	**n, cells**	**n, mice**
**WT-inj**	32	3	44	3	33	3
**WT-non**	35		38		33	
**KCC2-inj**	34	3	27	3	27	3
**KCC2-non**	35		27		33	

Statistics Summary: Figure 3.

	3A				3B			
2-way repeated measures ANOVA	**F (DFn, DFd)**		**p-value**	**significance**	**F (DFn, DFd)**		**p-value**	**significance**
**time x cohort**	F (8, 80) = 0.9524		0.4790	ns	F (8, 152) = 3.475		0.0010	**
**time**	F (4.856, 48.56) = 1.167		0.3389	ns	F (6.367, 121.0) = 19.08		<0.0001	n/a
**cohort**	F (1, 10) = 0.4249		0.5292	ns	F (1, 19) = 5.794		0.0264	n/a
**subject**	F (10, 80) = 5.977		<0.0001	****	F (19, 152) = 5.520		<0.0001	****
Post hoc Bonferroni multiple comparisons test	**Wild-Type**		**CaMKII-KCC2**		**Wild-Type**		**CaMKII-KCC2**	
	**adjusted p-value**	**significance**	**adjusted p-value**	**significance**	**adjusted p-value**	**significance**	**adjusted p-value**	**significance**
**pre vs 1-day post-SNC**	>0.9999	ns	>0.9999	ns	<0.0001	****	<0.0001	****
**pre vs 3-days post-SNC**	>0.9999	ns	>0.9999	ns	0.0003	***	0.0005	***
**pre vs 7-days post-SNC**	>0.9999	ns	0.5685	ns	0.0179	*	0.0022	**
**pre vs 14-days post-SNC**	>0.9999	ns	>0.9999	ns	0.0160	*	0.0017	**
**pre vs 21-days post-SNC**	>0.9999	ns	>0.9999	ns	0.1036	ns	0.0034	**
**pre vs 28-days post-SNC**	>0.9999	ns	>0.9999	ns	0.1117	ns	0.0018	**
**pre vs 35-days post-SNC**	>0.9999	ns	>0.9999	ns	0.0904	ns	0.0052	**
**pre vs 42-days post-SNC**	>0.9999	ns	>0.9999	ns	0.0713	ns	0.0004	***
Sample size	**Wild-Type**		**CaMKII-KCC2**		**Wild-Type**		**CaMKII-KCC2**	
**n, mice**	6		6		10		11	

		3C	3D
Mann Whitney test **Wild-Type vs CaMKII-KCC2**	**p-value**	0.8182	0.0079
	**significance**	ns	**
Sample size (same as 3A & 3B, respectively) **n, mice**	**Wild-Type**	6	10
	**CaMKII-KCC2**	6	11

Statistics Summary: Figure 4.

4D				
2-way repeated measures ANOVA	**F (DFn, DFd)**		**p-value**	**significance**
**drug x inj/non**	F (8, 80) = 2.358		0.0248	*
**drug**	F (3.852, 38.52) = 10.64		<0.0001	n/a
**inj/non**	F (1, 10) = 1.585		0.2367	n/a
**subject**	F (10, 80) = 8.857		<0.0001	****
Post hoc Bonferroni multiple comparisons test	**Saline**		**Bumetanide**	
	**adjusted p-value**	**significance**	**adjusted p-value**	**significance**
**pre vs 1-day post-SNC**	0.0171	*	0.0057	**
**pre vs 3-days post-SNC**	0.0046	**	0.0255	*
**pre vs 7-days post-SNC**	0.0555	ns	0.0040	**
**pre vs 14-days post-SNC**	>0.9999	ns	0.0232	*
**pre vs 21-days post-SNC**	>0.9999	ns	0.0142	*
**pre vs 28-days post-SNC**	>0.9999	ns	0.0014	**
**pre vs 35-days post-SNC**	0.1252	ns	0.0690	ns
**pre vs 42-days post-SNC**	0.6675	ns	0.0043	**
Sample size	**Saline**		**Bumetanide**	
**n, mice**	6		6	

4E		
Mann Whitney test **Saline vs Bumetanide**	**p-value**	0.0087
	**significance**	**
Sample size (same as 4D) **n, mice**	**Saline**	6
	**Bumetanide**	6

Statistics Summary: Figure 5.

**5B**			
2-way repeated measures ANOVA	**F (DFn, DFd)**	**p-value**	**significance**
**genotype x inj/non**	F (1, 49) = 0.01593	0.9001	ns
**genotype**	F (1, 49) = 2.555	0.1164	ns
**inj/non**	F (1, 49) = 41.45	<0.0001	****
**subject**	F (49, 49) = 6.264	<0.0001	****

Post hoc Bonferroni multiple comparisons test		**adjusted p-value**	**significance**
**non vs inj**	**Wild-Type**	<0.0001	****
	**CaMKII-KCC2**	<0.0001	****

Sample size	**n, L4-L5 spinal cord ventral horn sections**	**n, mice**
**Wild-Type (inj)**	25	3
**Wild-Type (non)**	25	
**CaMKII-KCC2 (inj)**	26	3
**CaMKII-KCC2 (non)**	26	

**5C**		
Mann Whitney test **Wild-Type vs CaMKII-KCC2**	**p-value**	0.6844
	**significance**	ns
Sample size **n, [spinal cord sections; mice]**	**Wild-Type**	[25; 3]
	**CaMKII-KCC2**	[26; 3]

Statistics Summary: Figure 6.

6B						
2-way repeated measures ANOVA	**F (DFn, DFd)**				**p-value**	**significance**
**time x cohort**	F (6, 36) = 9.939				<0.0001	****
**time**	F (1.944, 23.32) = 139.3				<0.0001	n/a
**cohort**	F (2, 12) = 12.29				0.0012	n/a
**subject**	F (12, 36) = 0.5369				0.8759	ns
Post hoc Bonferroni multiple comparisons test	**WT-SNC**		**KCC2-SNC**		**WT-CUT**	
	**adjusted p-value**	**significance**	**adjusted p-value**	**significance**	**adjusted p-value**	**significance**
**pre vs 1-day post-SNC**	<0.0001	****	0.0001	***	0.0121	*
**pre vs 7-days post-SNC**	<0.0001	****	<0.0001	****	0.0056	**
**pre vs 42-days post-SNC**	0.3261	ns	0.5504	ns	0.0194	*
Sample size	**WT-SNC**		**KCC2-SNC**		**WT-CUT**	
**n, mice**	6		6		3	

6C		
Kruskal-Wallis test	**p-value**	**significance**
	0.0174	*
Post hoc Dunn's multiple comparisons test	**adjusted p-value**	**significance**
**WT-SNC vs KCC2-SNC**	>0.9999	ns
**WT-SNC vs WT-CUT**	0.0196	*
Sample size (same as 6B)	**n, mice**	
**WT-SNC**	6	
**KCC2-SNC**	6	
**WT-CUT**	3	

Statistics Summary: Figure 7.

	7C: a		7C: b		7D: a		7D: b	
Kruskal-Wallis test	**p-value**	**significance**	**p-value**	**significance**	**p-value**	**significance**	**p-value**	**significance**
	0.5091	ns	<0.0001	****	0.2924	ns	<0.0001	****
Post hoc Dunn's multiple comparisons test	**adjusted p-value**	**significance**	**adjusted p-value**	**significance**	**adjusted p-value**	**significance**	**adjusted p-value**	**significance**
**WT-non vs WT-inj**	>0.9999	ns	<0.0001	****	>0.9999	ns	<0.0001	****
**KCC2-non vs KCC2-inj**	0.7339	ns	<0.0001	****	0.3498	ns	0.6786	ns
**WT-non vs KCC2-non**	>0.9999	ns	0.0596	ns	0.3693	ns	0.3878	ns
**WT-inj vs KCC2-inj**	>0.9999	ns	0.4625	ns	>0.9999	ns	<0.0001	****
Sample size	**n, cells**	**n, mice**	**n, cells**	**n, mice**	**n, cells**	**n, mice**	**n, cells**	**n, mice**
**WT-inj**	57	3	68	3	46	3	68	3
**WT-non**	65		54		56		62	
**KCC2-inj**	47	3	47	3	50	3	61	3
**KCC2-non**	47		52		43		52	

Statistics Summary: Figure 8.

	8D		8E	
Kruskal-Wallis test	**p-value**	**significance**	**p-value**	**significance**
	<0.0001	****	0.0001	***
Post hoc Dunn's multiple comparisons test	**adjusted p-value**	**significance**	**adjusted p-value**	**significance**
**WT-non vs WT-inj**	0.0065	**	0.0003	***
**KCC2-non vs KCC2-inj**	0.0062	**	>0.9999	ns
**WT-non vs KCC2-non**	>0.9999	ns	0.9465	ns
**WT-inj vs KCC2-inj**	>0.9999	ns	0.0009	***
Sample size	**n, cells**	**n, mice**	**n, cells**	**n, mice**
**Saline-inj**	46	3	62	3
**Saline-non**	51		61	
**Bic-inj**	35	3	53	3
**Bic-non**	33		46	

8F				
2-way repeated measures ANOVA	**F (DFn, DFd)**		**p-value**	**significance**
**drug x inj/non**	F (8, 80) = 5.856		<0.0001	****
**drug**	F (4.072, 40.72) = 20.77		<0.0001	n/a
**inj/non**	F (1, 10) = 2.862		0.1215	n/a
**subject**	F (10, 80) = 23.12		<0.0001	****
Post hoc Bonferroni multiple comparisons test	**Saline**		**Bicuculline**	
	**adjusted p-value**	**significance**	**adjusted p-value**	**significance**
**pre vs 1-day post-SNC**	0.0014	**	0.0308	*
**pre vs 3-days post-SNC**	0.0081	**	0.0082	**
**pre vs 7-days post-SNC**	0.0275	*	0.0157	*
**pre vs 14-days post-SNC**	0.3340	ns	0.0065	**
**pre vs 21-days post-SNC**	0.0028	**	0.0261	*
**pre vs 28-days post-SNC**	>0.9999	ns	0.0248	*
**pre vs 35-days post-SNC**	>0.9999	ns	0.0023	**
**pre vs 42-days post-SNC**	0.6453	ns	0.0043	**
Sample size	**Saline**		**Bicuculline**	
**n, mice**	6		6	

8G		
Mann Whitney test **Wild-Type vs CaMKII-KCC2**	**p-value**	0.0152
	**significance**	*
Sample size (same as 8F) **n, mice**	**Saline**	6
	**Bumetanide**	6

Supplementary Figure 1

A

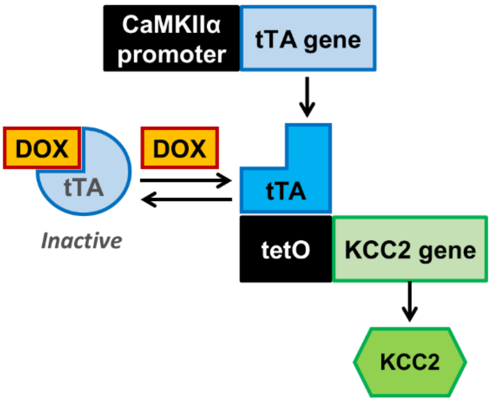

B

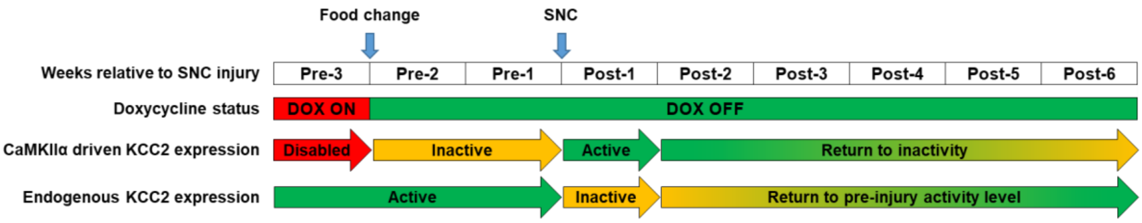

Schematics summarizing the strategy used to selectively prevent injury induced KCC2 downregulation in motoneurons.

A. In CaMKII-KCC2 mice, the tetO-tTA conditional expression system additionally couples KCC2 expression to the CaMKIIα promoter. Here, the tetracycline transactivator (tTA) gene is knocked-in downstream of the CaMKIIα promoter and the tetracycline response element (tetO) promoter is knocked-in upstream of the KCC2 gene. Thus, CaMKIIα driven tTA expression results in KCC2 expression. This gene expression setup can be reversibly disabled by doxycycline (DOX) as DOX-tTA binding prevents tTA-TRE binding.

B. CaMKIIα expression in healthy mature motoneurons is normally very low but dramatically increases after injury. Thus, CaMKIIα driven KCC2 expression in motoneurons remains inactive after switching CaMKII-KCC2 mice from on-doxycycline (DOX ON) to off-doxycycline (DOX OFF), but becomes active after subsequent motoneuron injury. In this way, CaMKIIα driven KCC2 expression selectively compensates for injury induced KCC2 downregulation in motoneurons.

Supplementary Figure 2

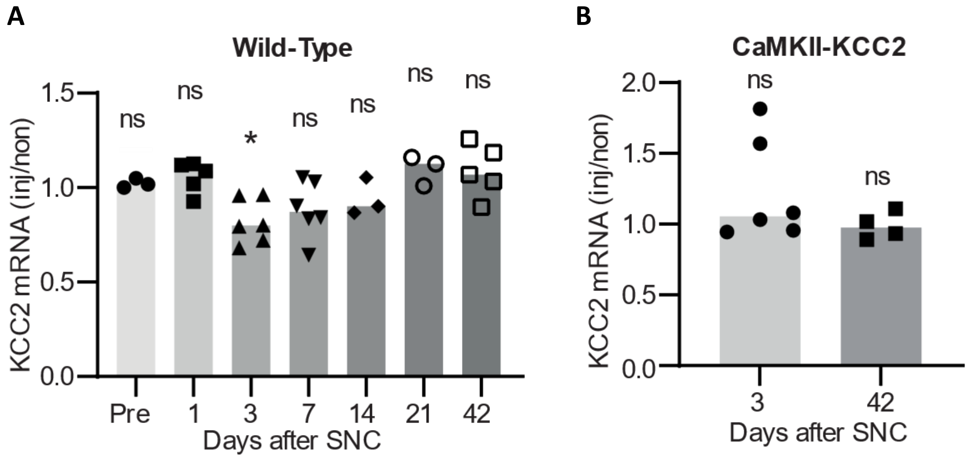

KCC2 mRNA expression in the spinal cord ventral horn measured by RT-qPCR before and at various time-points after SNC.

KCC2 mRNA expression (normalized to GAPDH) in the L4-L5 ventral horns is plotted as a ratio between the injured-side ventral horn (inj) and uninjured-side ventral (non), i.e. (inj/non). Various time-points before (pre) and after SNC are examined. Each data point represents one mouse. Bars indicate cohort medians. *p <0.05; one-sample Wilcoxon test, cohort medians compared to a hypothetical value of 1.0, i.e. identical KCC2 mRNA expression between injured-side and uninjured-side.

A. Wild-type cohorts at [pre, 1, 3, 7, 14, 21, 42]-days post-SNC, n = [3, 5, 6, 6, 3, 3, 5] mice.

B. CaMKII-KCC2 cohorts at [3, 42]-days post-SNC, n = [6, 4] mice.

	1-sample Wilcoxon test	**Days after SNC**						
		**Pre**	**1**	**3**	**7**	**14**	**21**	**42**
**s2A**	**p-value**	0.5000	0.1875	0.0313	0.1563	0.5000	0.2500	0.3125
	**significance**	ns	ns	*	ns	ns	ns	ns
	***Sample size* n, mice**	3	5	6	6	3	3	5
**s2B**	**p-value**	n/a	n/a	n/a	0.3125	n/a	n/a	>0.9999
	**significance**	n/a	n/a	n/a	ns	n/a	n/a	ns
	***Sample size* n, mice**	n/a	n/a	n/a	6	n/a	n/a	4

Because characterization of KCC2 expression by immunohistochemistry is highly labour intensive, RT-qPCR was used as a preliminary screening approach to suggest a time-point where SNC-induced KCC2 expression downregulation would be most pronounced. Based on these results, the 3-days post-SNC time-point was selected as a time-point for the KCC2 immunohistochemistry experiments (Fig. 2 main text). RT-qPCR was also used to preliminarily check that SNC-induced KCC2 expression downregulation was selectively prevented in CaMKII-KCC2 mice. Note that the RT-qPCR data reflects KCC2 mRNA expression from all cell-types (not motoneurons exclusively) in the spinal cord ventral horn.

Supplementary Figure 3

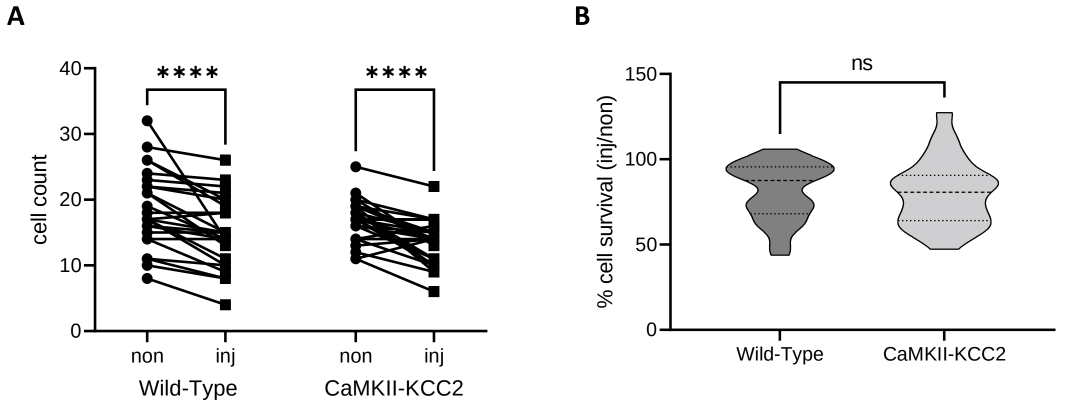

Survival of all motoneuron types at 42-days post-SNC.

Figures 5B-5C in the main text quantify the survival rates of alpha-motoneurons (ChAT-positive, NeuN-positive cells in ventral horn Rexed lamina IX). Here, the survival rates of all motoneuron types (ChAT-positive cells in ventral horn Rexed lamina IX) are quantified from the same spinal cord sections.

A. Motoneuron cell counts at 42-days post-SNC in the injured-side (inj) and uninjured-side (non) ventral horn (L4-L5, Rexed lamina IX), for wild-type and CaMKII-KCC2 mice. Each pair of connected data points plots injured-side (inj) and uninjured-side (non) cell counts per one spinal cord section. *p <0.05, **p <0.01, ***p <0.001, ****p <0.0001; 2-way repeated measures ANOVA, post hoc Bonferroni multiple comparisons test. Cohorts [wild-type, CaMKII-KCC2], n = [25, 26] sections, 3 wild-type, 3 CaMKII-KCC2 mice.

**Supplementary 3A**			
2-way repeated measures ANOVA	**F (DFn, DFd)**	**p-value**	**significance**
**genotype x inj/non**	F (1, 49) = 0.0004508	0.9831	ns
**genotype**	F (1, 49) = 2.180	0.1462	ns
**inj/non**	F (1, 49) = 48.97	<0.0001	****
**subject**	F (49, 49) = 5.417	<0.0001	****

Post hoc Bonferroni multiple comparisons test		**adjusted p-value**	**significance**
**non vs inj**	**Wild-Type**	<0.0001	****
	**CaMKII-KCC2**	<0.0001	****

B. Truncated violin plots quantifying normalized motoneuron survival ([injured-side/uninjured-side] cell count x 100%) in the injured-side ventral horn (L4-L5, Rexed lamina IX), for wild-type and CaMKII-KCC2 mice at 42-days post-SNC. Cohorts as in Supplementary Figure 2A. *p < 0.05; Mann Whitney test.

**Supplementary 3B**		
Mann Whitney test	**p-value**	**significance**
**Wild-Type vs CaMKII-KCC2**	0.5779	ns

Supplementary Figure 4

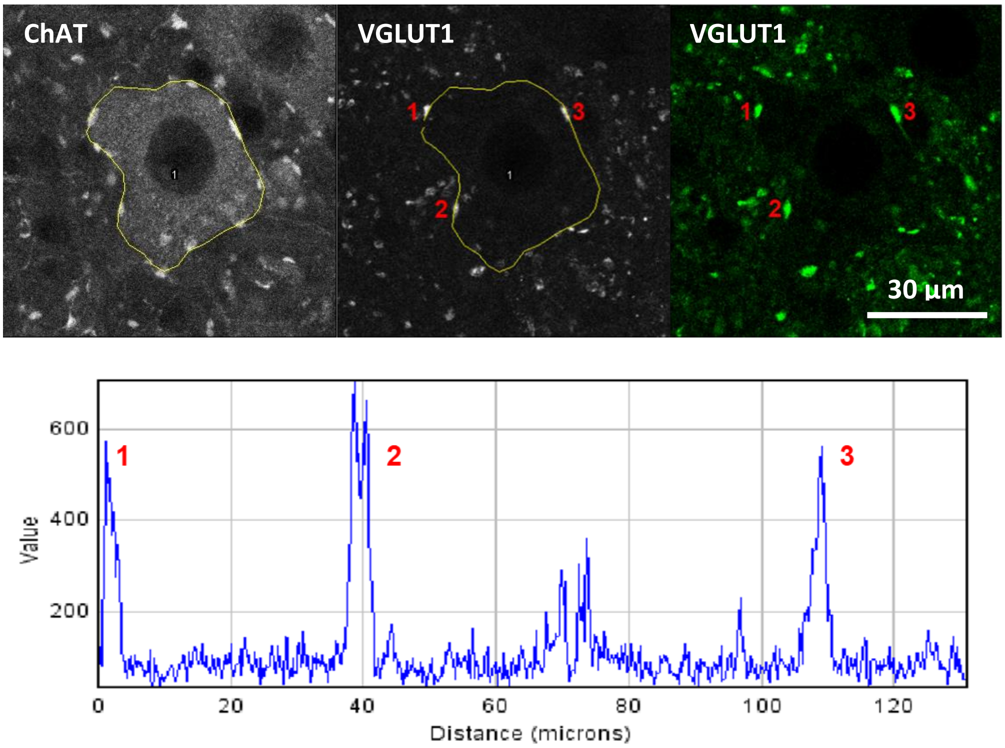

Quantification of VGLUT1-positive and GAD67-positive terminals

VGLUT1-positive and GAD67-positive terminals synapsing onto motoneuron somas could be resolved as distinct punctae along the soma perimeter. To quantify these, the soma perimeter as demarcated by ChAT immunofluorescence was traced (top left panel) to generate a line plot profile of VGLUT1 or GAD67 immunofluorescence intensity (top middle and right panels).

From this fluorescence intensity plot (bottom panel), peaks larger than 2x the baseline and wider than 2 µm were counted as individual VGLUT1/GAD67-positive terminals. To account for variations in motoneuron size, the VGLUT1/GAD67-positive terminal counts were divided by the motoneuron soma perimeter length and expressed as terminal density per 100 µm of soma perimeter.

Supplementary Figure 5

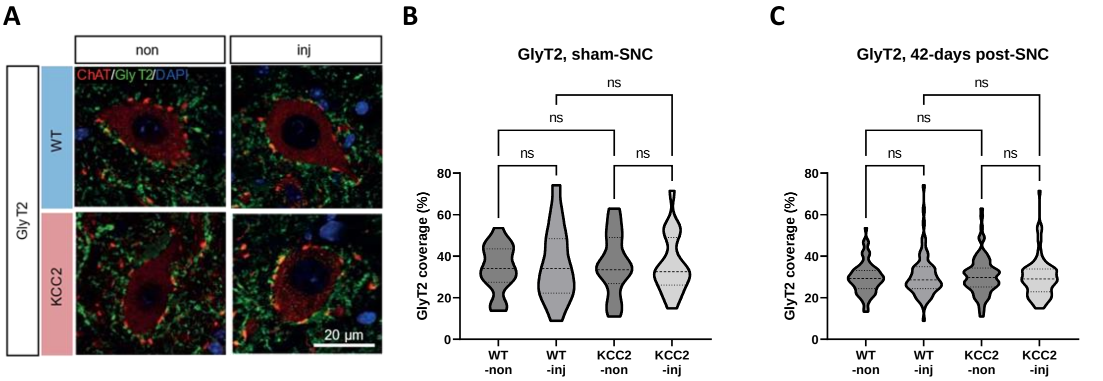

Quantification of GlyT2-positive terminals

GlyT2-positive terminals synapsing onto motoneuron somas were unable to be resolved as distinct punctae and were instead quantified as the (%) area coverage of soma perimeter ROI.

A. Representative motoneurons from the injured-side (inj) and uninjured-side (non) ventral horn (L4-L5, Rexed lamina IX), for wild-type (WT) and CaMKII-KCC2 (KCC2) mice at 42-days post-SNC. Immunofluorescence for GlyT2 (green), ChAT (red) and DAPI (blue). Scale bar, 20 µm. GlyT2 terminals cannot be individually resolved and appear as a green border along the red soma perimeter.

B, C. Truncated violin plots quantifying plasmalemmal GlyT2 immunofluorescence (% ROI coverage), for individual motoneuron somas from the injured-side (inj) and uninjured-side (non) ventral horn (L4-L5, Rexed lamina IX), for wild-type (WT-inj, WT-non), and CaMKII-KCC2 (KCC2-inj, KCC2-non), mice at 42-days after SNC or sham-SNC. *p <0.05; Kruskal-Wallis test, post-hoc Dunn's multiple comparisons test. Cohorts [WT-inj, WT-non, KCC2-inj, KCC2-non]. **(B)** 42-days after sham-SNC, n = [16, 18, 19, 17] cells, 2 wild-type, 2 CaMKII-KCC2 mice. **(C)** 42-days post-SNC, n = [74, 79, 85, 87] cells, 4 wild-type, 4 CaMKII-KCC2 mice.

	**Supplementary 5B**		**Supplementary 5C**	
Kruskal-Wallis test	**p-value**	**significance**	**p-value**	**significance**
	0.9770	ns	0.8185	ns
Post hoc Dunn's multiple comparisons test	**adjusted p-value**	**significance**	**adjusted p-value**	**significance**
**WT-non vs WT-inj**	>0.9999	ns	>0.9999	ns
**KCC2-non vs KCC2-inj**	>0.9999	ns	>0.9999	ns
**WT-non vs KCC2-non**	>0.9999	ns	>0.9999	ns
**WT-inj vs KCC2-inj**	>0.9999	ns	>0.9999	ns

Supplementary Figure 6

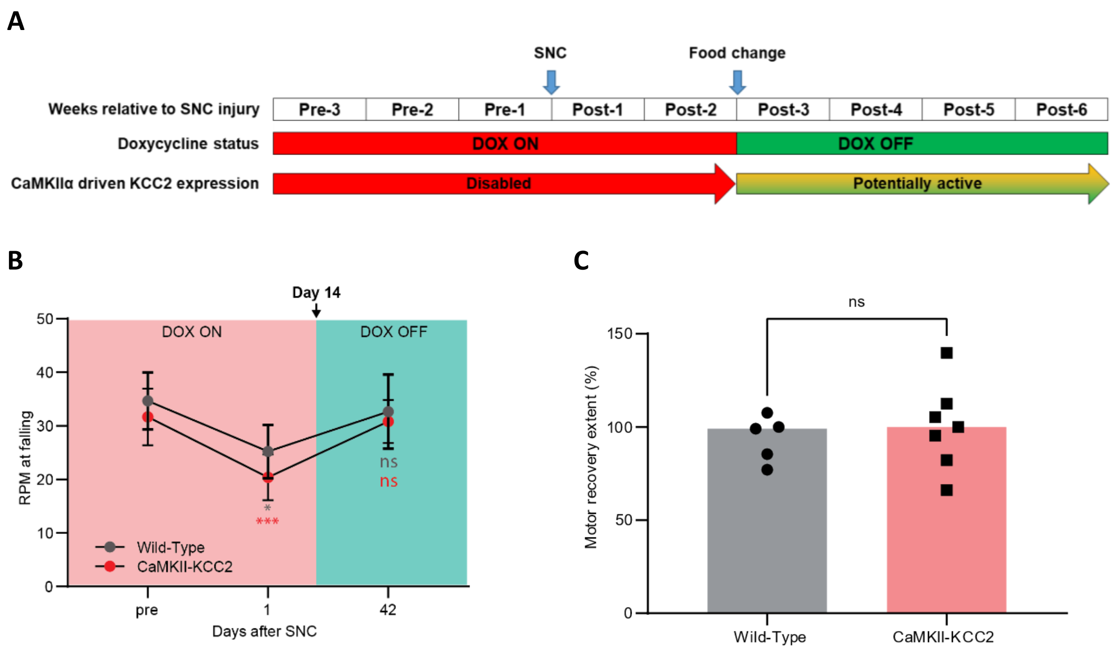

KCC2 downregulation affects motor function recovery during the early post-injury period.

Impaired motor function recovery in CaMKII-KCC2 mice is attributed to the prevention of injury induced KCC2 downregulation during the early post-injury period (Fig. 2 main text; Supp. Fig. 1). However, subtle KCC2 overexpression in the late post-injury period is a potential alternative contributor to impaired motor function recovery. One approach for ruling this out is to delay the on-doxycycline (DOX ON) to off-doxycycline (DOX OFF) switch to the late post-injury period. If subtle KCC2 overexpression in the late post-injury meaningfully contributes to impaired motor function recovery, CaMKII-KCC2 mice should still have impaired motor function recovery compared to wild-type mice under this delayed doxycycline schedule.

A. In a subset of wild-type and CaMKII-KCC2 mice, the on-doxycycline (DOX ON) to off-doxycycline (DOX OFF) switch was delayed from 2-3 weeks before SNC to 14-days post-SNC.

B. Motor performance scores (rpm at falling), measured before (pre), and at 1 and 42-days after SNC, for wild-type (WT) and CaMKII-KCC2 (KCC2) mice, with both cohorts on-doxycycline until 14-days post-SNC. Cohort means and standard deviations are plotted. Cohorts [wild-type, CaMKII-KCC2], n = [5, 7] mice. *$p < 0.05$; 2-way repeated measures ANOVA, post hoc Bonferroni multiple comparisons test for pre vs [1, 42]-days post-SNC per each cohort.

Supplementary 6B				
2-way repeated measures ANOVA	**F (DFn, DFd)**		**p-value**	**significance**
**time x cohort**	F (2, 20) = 0.5004		0.6137	ns
**time**	F (1.711, 17.11) = 28.37		<0.0001	****
**cohort**	F (1, 10) = 1.749		0.2154	ns
**subject**	F (10, 20) = 4.001		0.0040	**
Post hoc Bonferroni multiple comparisons test	**Wild-Type**		**CaMKII-KCC2**	
	**adjusted p-value**	**significance**	**adjusted p-value**	**significance**
**pre vs 1-day post-SNC**	0.0160	*	0.0006	***
**pre vs 42-days post-SNC**	0.6091	ns	>0.9999	ns

C. Degree of motor function recovery ([day-42/pre] rpm x100%), for wild-type (WT) and CaMKII-KCC2 (KCC2) mice after SNC, both cohorts on doxycycline until 14-days post-SNC. Each data point represents one mouse. Bars indicate cohort medians. Cohorts as in Supplementary Figure 5B. *p < 0.05, Mann Whitney test.

Supplementary 6C		
Mann Whitney test	**p-value**	**significance**
**WT vs KCC2**	0.6717	ns

Supplementary Figure 7

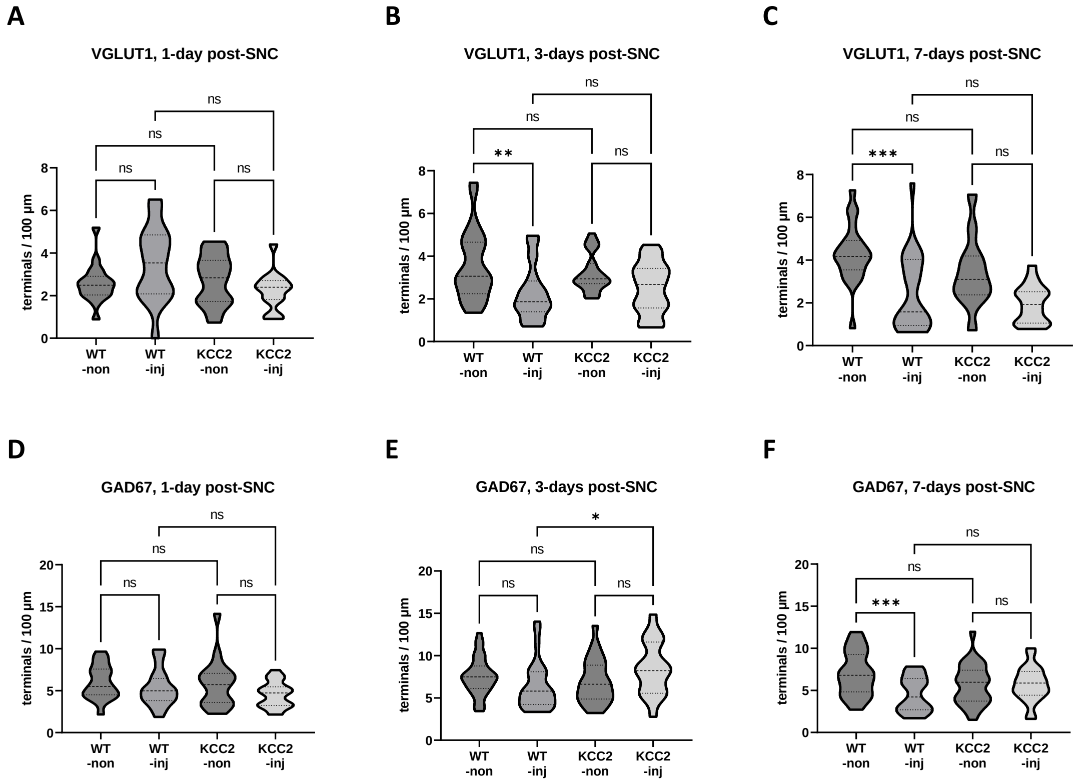

Reorganization of VGLUT1 and GAD67 synaptic inputs to motoneurons during the early post-injury period.

The causal relationship between injury induced KCC2 downregulation and motor function recovery is correlated with the long-term reorganization of GAD67 (but not VGLUT1) synaptic inputs to motoneurons (Fig. 7 main text). At the same time, injury induced KCC2 downregulation is limited to the early post-injury period. Thus, the most prominent features of these long-term synaptic reorganization trends should start to appear during the early post-injury period. To test this, VGLUT1 and GAD67 terminals were quantified at 1, 3 and 7-days post-SNC in wild-type and CaMKII-KCC2 mice.

A-F. Truncated violin plots quantifying the number of VGLUT1 (A-C), and GAD67 (D-F), positive terminals (count per 100 µm), for individual motoneuron somas from the injured-side (inj) and uninjured-side (non) ventral horn (at L4-L5, Rexed lamina IX), for wild-type (WT-inj, WT-non), and CaMKII-KCC2 (KCC2-inj, KCC2-non), mice at various time-points after SNC. *p <0.05, **p <0.01, ***p <0.001, ***p <0.0001; Kruskal-Wallis test, post-hoc Dunn's multiple comparisons test.

(A-C) Cohorts [WT-inj, WT-non, KCC2-inj, KCC2-non]. VGLUT1 terminal count at: (A) 1-day post-SNC, n = [16, 18, 15, 18] cells, 3 wild-type, 3 CaMKII-KCC2 mice; (B) 3-days post-SNC, n = [27, 26, 30, 28] cells, 3 wild-type, 3 CaMKII-KCC2 mice; (C) 7-days post-SNC, n = [25, 22, 17, 12] cells, 3 wild-type, 3 CaMKII-KCC2 mice.

	**Supplementary 7A**		**Supplementary 7B**		**Supplementary 7C**	
Kruskal-Wallis test	**p-value**	**significance**	**p-value**	**significance**	**p-value**	**significance**
	0.1263	ns	0.0013	**	<0.0001	****
Post hoc Dunn's multiple comparisons test	**adjusted p-value**	**significance**	**adjusted p-value**	**significance**	**adjusted p-value**	**significance**
**WT-non vs WT-inj**	0.4959	ns	0.0041	**	0.0004	***
**KCC2-non vs KCC2-inj**	0.9172	ns	0.1196	ns	0.1214	ns
**WT-non vs KCC2-non**	>0.9999	ns	>0.9999	ns	0.3539	ns
**WT-inj vs KCC2-inj**	0.0742	ns	>0.9999	ns	>0.9999	ns

(D-F) Cohorts [WT-inj, WT-non, KCC2-inj, KCC2-non]. GAD67 terminal count at: (D) 1-day post-SNC, n = [19, 18, 20, 18] cells, 3 wild-type, 3 CaMKII-KCC2 mice; (E) 3-days post-SNC, n = [28, 33, 18, 25] cells, 3 wild-type, 3 CaMKII-KCC2 mice; (F) 7-days post-SNC, n = [31, 35, 23, 28] cells, 3 wild-type, 3 CaMKII-KCC2 mice.

	**Supplementary 7D**		**Supplementary 7E**		**Supplementary 7F**	
Kruskal-Wallis test	**p-value**	**significance**	**p-value**	**significance**	**p-value**	**significance**
	0.1573	ns	0.0366	*	0.0026	**
Post hoc Dunn's multiple comparisons test	**adjusted p-value**	**significance**	**adjusted p-value**	**significance**	**adjusted p-value**	**significance**
**WT-non vs WT-inj**	>0.9999	ns	0.1232	ns	0.0006	***
**KCC2-non vs KCC2-inj**	0.4635	ns	0.3734	ns	>0.9999	ns
**WT-non vs KCC2-non**	>0.9999	ns	>0.9999	ns	0.3026	ns
**WT-inj vs KCC2-inj**	>0.9999	ns	0.0313	*	0.2331	ns

Supplementary Figure 8

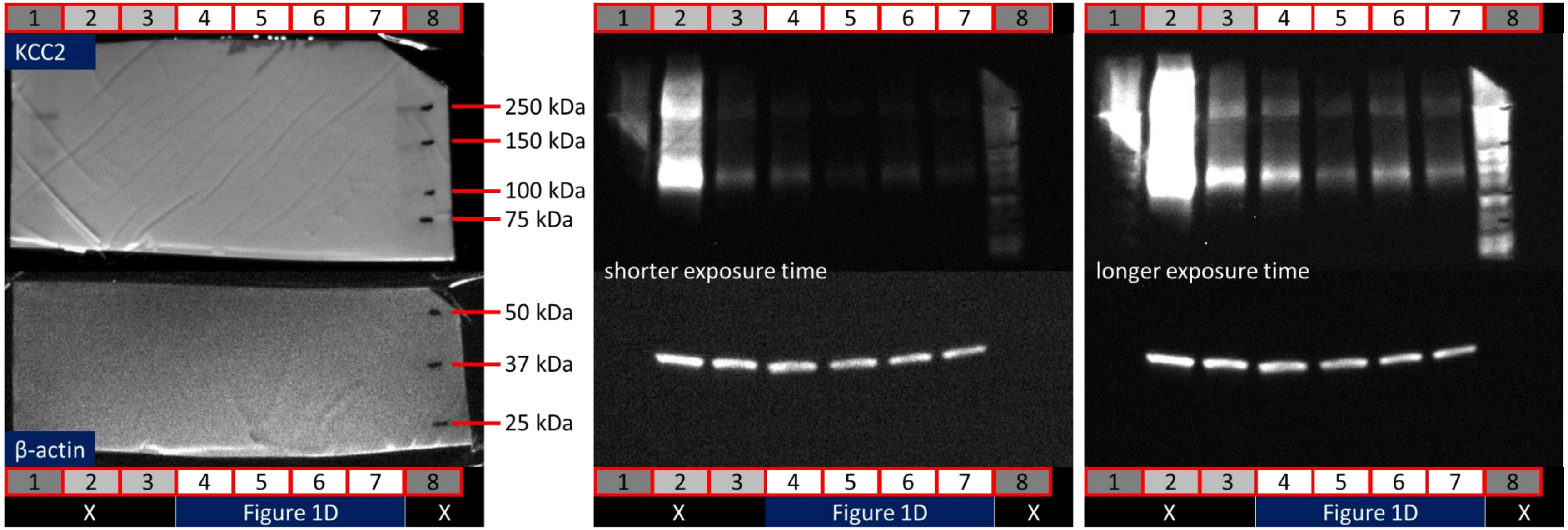

Full length western blot corresponding to Figure 1D in the main text.

Figure 1D in the main text displays cropped KCC2 and β-actin protein bands which correspond to lanes 4-7 of the full length western blot shown here. In this western blot, proteins samples were extracted from the injured-side (inj) and uninjured-side (non) L4-L5 spinal cord ventral horn of an individual wild-type mouse (WT-inj, WT-non) and an individual CaMKII-KCC2 mouse (KCC2-inj, KCC2-non) at 3-days post-SNC. Protein samples were also extracted from the brains of these mice as an additional positive control. Precision Plus Protein WesternC Standards (BioRad, #161-0376) were used for the protein size ladder. The identity of the lanes are as follows: [1, 2, 3, **4, 5, 6, 7,** 8] = [ladder, KCC2 (brain), WT (brain), **WT-non, WT-inj, KCC2-non, KCC2-inj,** ladder].

As shown in the left panel, the western blot membrane was cut into two sections between the 75 kDa and 50 kDa size markers. The 75-250 kDa and 25-50 kDa membrane sections were separately incubated with the primary antibodies for KCC2 and β-actin respectively.

As shown in the middle and right panels, protein bands were visualized by chemiluminescence and imaged at shorter and longer exposure times respectively. Image brightness and contrast in both panels have been auto-adjusted in ImageJ. Fluorescent bands corresponding to KCC2 (~140 kDa glycosylated monomer, ~300 kDa SDS resistant non-dissociated dimer) and β-actin (42 kDa) are visible in all sample lanes (2-7).

At the time of sample collection, all mice had been off-doxycycline for approximately 3 weeks. As expected, this resulted in dramatic KCC2 overexpression in the CaMKII-KCC2 brain sample (middle panel, shorter exposure time) – see Goulton et al., 2018. Also as expected, KCC2 expression levels in all spinal cord samples were comparable to the wild-type brain sample, albeit slightly lower (right panel, longer exposure time) – see Supplementary Figure 1. Fluorescence quantification of the KCC2 bands, normalized against the corresponding β-actin bands, indicated that ventral horn KCC2 expression at 3-days post-SNC was significantly lower in injured-side vs uninjured-side in wild-type mice but similar between both sides for CaMKII-KCC2 mice – see Figure 1D in the main text.

Note that in addition to cropping, the western blot image shown here (right panel) has been grayscale inverted and rotated to be straight in Figure 1D in the main text.

Supplementary Figure 9

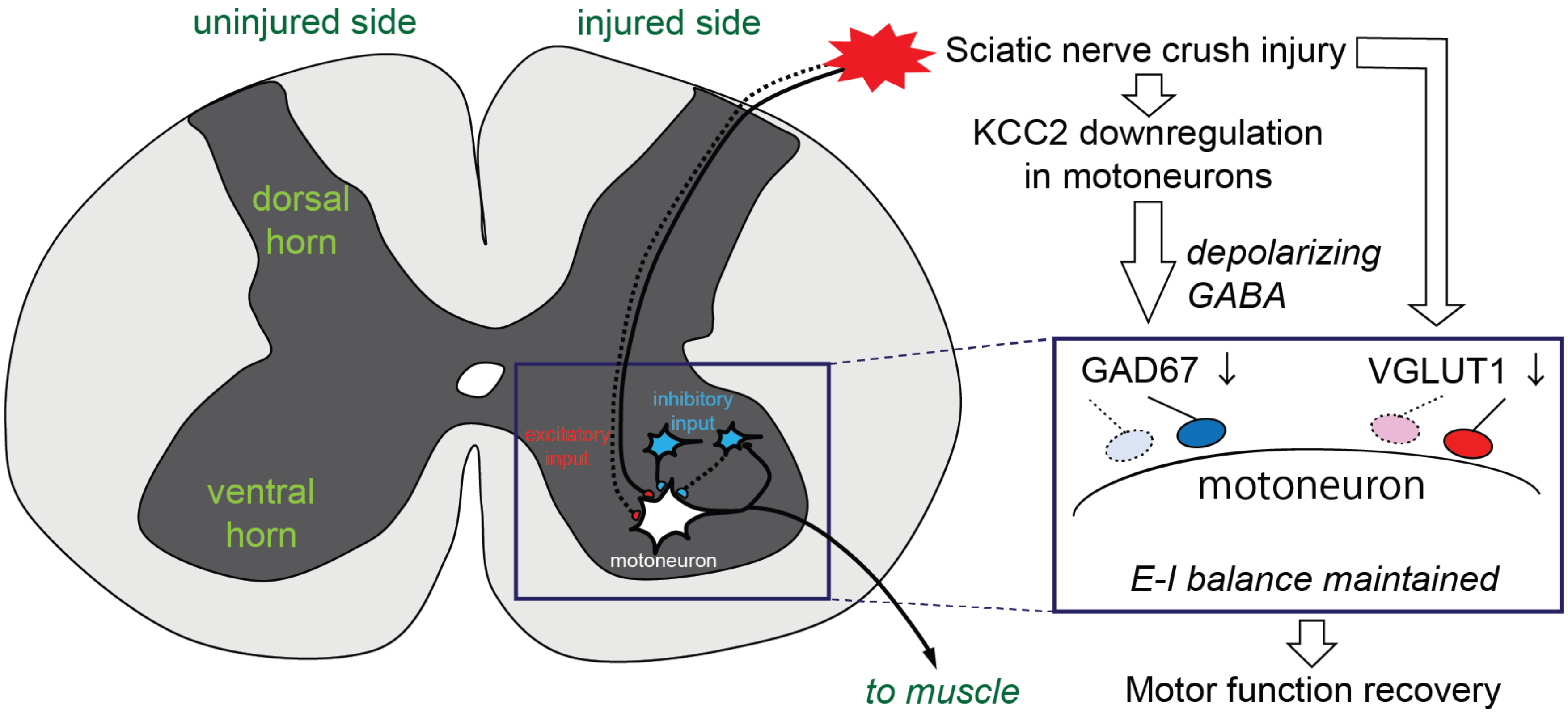

Schematic of how injury-induced KCC2 downregulation in spinal cord motoneurons promotes motor recovery.

Following SNC, various processes drive synaptic stripping of excitatory (VGLUT1) input to motoneurons. Separately, SNC induces KCC2 downregulation in motoneurons which causes GABAergic signalling to become depolarizing. This drives synaptic stripping of inhibitory (GAD67) input to motoneurons. Thus, the loss of excitatory input is adaptively matched by a loss of inhibitory input thereby maintaining appropriate excitatory-inhibitory (E-I) balance.
